# Identification of healthspan-promoting genes in *Caenorhabditis elegans* based on a human GWAS study

**DOI:** 10.1007/s10522-022-09969-8

**Published:** 2022-06-24

**Authors:** Nadine Saul, Ineke Dhondt, Mikko Kuokkanen, Markus Perola, Clara Verschuuren, Brecht Wouters, Henrik von Chrzanowski, Winnok H. De Vos, Liesbet Temmerman, Walter Luyten, Aleksandra Zečić, Tim Loier, Christian Schmitz-Linneweber, Bart P. Braeckman

**Affiliations:** 1 Molecular Genetics Group, Institute of Biology, Humboldt University of Berlin, Berlin, Germany; 2 Laboratory of Aging Physiology and Molecular Evolution, Biology Department, Ghent University, Ghent, Belgium; 3 Genomics and Biomarkers Unit, Department of Health, National Institute for Health and Welfare, Helsinki, Finland; 4 Department of Human Genetics and South Texas Diabetes and Obesity Institute, School of Medicine, The University of Texas Rio Grande Valley, Brownsville, TX USA; 5 Biology Department, KU Leuven, Leuven, Belgium; 6 The Mina & Everard Goodman Faculty of Life Sciences, Bar-Ilan University, Ramat-Gan, Israel; 7 Laboratory of Cell Biology and Histology, Department of Veterinary Sciences, University of Antwerp, Antwerp, Belgium

**Keywords:** Healthspan, Ageing, GWAS, *C. elegans*, Muscle integrity, Stress resistance

## Abstract

To find drivers of healthy ageing, a genome-wide association study (GWAS) was performed in healthy and unhealthy older individuals. Healthy individuals were defined as free from cardiovascular disease, stroke, heart failure, major adverse cardiovascular event, diabetes, dementia, cancer, chronic obstructive pulmonary disease (COPD), asthma, rheumatism, Crohn’s disease, malabsorption or kidney disease. Six single nucleotide polymorphisms (SNPs) with unknown function associated with ten human genes were identified as candidate healthspan markers. Thirteen homologous or closely related genes were selected in the model organism *C. elegans* for evaluating healthspan after targeted RNAi-mediated knockdown using pathogen resistance, muscle integrity, chemotaxis index and the activity of known longevity and stress response pathways as healthspan reporters. In addition, lifespan was monitored in the RNAi-treated nematodes. RNAi knockdown of *yap-1*, *wwp-1*, *paxt-1* and several *acdh* genes resulted in heterogeneous phenotypes regarding muscle integrity, pathogen resistance, chemotactic behaviour, and lifespan. Based on these observations, we hypothesize that their human homologues *WWC2*, *CDKN2AIP* and *ACADS* may play a role in health maintenance in the elderly.

**Supplementary Information:**

The online version contains supplementary material available at 10.1007/s10522-022-09969-8.

## Introduction

Ageing is a complex and so far unavoidable process to virtually all life (Martínez and Bridge 2012). In the past decades, lifespan measurements were the main basis for collecting ageing-relevant data of populations. However, the continuous increase in life expectancy, paired with an unchanged age of the onset of morbidity and ageing-associated diseases (Crimmins 2015) highlights that lifespan is not representative for the various phenotypes of ageing. Thus, the focus is increasingly on health and healthspan, defined by the lack of diseases and dysfunctions addressing physiological, physical, cognitive, and reproductive functions (Fuellen et al. 2019).

Efforts to uncover the genetic background of ageing contribute to finding means of maintaining health even into advanced old age. Genetic manipulations in model organisms with genetic similarity to humans, using gene-knockout strains, reporter-fusion transgenic lines, over-expressing strains or *via* RNA interference (RNAi), offer possibilities to examine the contribution of genes and pathways to ageing phenotypes (de Magalhães and Toussaint 2004; Hühne et al. 2014; Link 2001; Minois et al. 2010). However, screening all genes for ageing-relevance is a daunting challenge due to the sheer number of candidates and of different ageing-related phenotypes. Genome-wide association studies (GWAS) help filter and select candidate genes for in-depth investigation (Melzer et al. 2019).

In order to verify the impact of selected genes in the ageing process, a suitable model organism needs to be used that fulfils several criteria, as summarized by Lees et al. (2016). In our study, the nematode *Caenorhabditis (C.) elegans* was chosen owing to its numerous advantages as a model organism, such as short generation time, transparent body, easy and cheap maintenance, completely sequenced genome, ease of freezing transgenic and mutant strains for long-term storage, as well as well-established protocols and extensive libraries to perform RNAi-mediated gene silencing (Kamath and Ahringer 2003; Tissenbaum 2015). Biogerontologists particularly appreciate this worm due to its relatively high genetic concordance with humans in general (Kim et al. 2018), more specifically in terms of ageing-related human diseases (Markaki and Tavernarakis 2020). *C. elegans* also displays numerous human-like ageing phenotypes, including the decrease of locomotor abilities and muscle function (known as sarcopenia), the decline of sensory and cognitive capacities, and a weakened defence against pathogens (Bulterijs and Braeckman 2020; Son et al. 2019).

This study aimed at identifying potential healthspan modulators with the aid of the human FINRISK data set (Borodulin et al. 2018). Single nucleotide polymorphisms (SNPs) associated with health maintenance were determined by comparing genomes from a healthy elderly (≥ 75 years) cohort group, that was free from chronic diseases, with an unhealthy elderly control group in two GWAS studies. Unhealthy participants were defined as being diagnosed with cardiovascular disease, stroke, heart failure, major adverse cardiovascular event, diabetes, dementia, cancer, chronic obstructive pulmonary disease (COPD), asthma, rheumatism, Crohn’s disease, malabsorption, or kidney disease. Worm homologues of genes with the smallest distance to those SNPs were selected, and their function was evaluated *via* RNA interference in *C. elegans* of different ages (3rd, 7th, and 14th day of adulthood) by analysing selected healthspan parameters. The selection of phenotypes is crucial to get an appropriate overview of the overall healthspan effect of a gene knockdown. Some of the most problematic and feared symptoms of ageing in humans are the loss of mobility and muscle strength (McGregor et al. 2014), the decline of cognitive and sensory function (Juan and Adlard 2019; Li and Lindenberger 2002) and the implications of immunosenescence as underlying cause for morbidity, mortality and numerous age-related diseases (Agarwal and Busse 2010; Barbé-Tuana et al. 2020). Thus, to cover these most relevant features of ageing, chemotactic perception, muscle integrity, as well as the activation of common stress pathways and pathogenic stress resistance were chosen as representative phenotypes in *C. elegans*. In addition, the effects of the RNAi-treatments on lifespan were determined. This way, we identified at least three genes with a significant contribution to healthy ageing.

## Methods

### Genome-wide association study

We performed two genome-wide association studies using Finnish FINRISK population surveys from the years 1992, 1997, 2002 and 2007. At first (GWAS1), we compared the genomes of healthy individuals (n = 750), who had reached a minimum age of 75 years, to individuals who were unhealthy survivors—75 years old or older (n = 1502). Secondly (GWAS2), to get more power, we tested for association of the same set of healthy individuals (n = 750) and unhealthy survivors as in GWAS1, but added individuals with all-cause mortality under 75 years of age to the analysis (n = 2663). We used national registers to assess the health of FINRISK participants. We scanned medical reimbursement records and purchased data including death registers and the hospital discharge registers for cardiovascular disease, stroke (including intracerebral haemorrhage), heart failure, major adverse cardiovascular event, diabetes, dementia, any cancer (abnormal cells divide and invade nearby tissues, neoplasms), chronic obstructive pulmonary disease (COPD), asthma, rheumatism, Crohn’s disease, malabsorption (intestinal malabsorption, postprocedural disorders of the digestive system not elsewhere classified, celiac disease & digestive system disorders) or chronic or unspecified kidney disease (Online Resource 1). Logistic regressions were adjusted by age, sex, genotyping batches, healthcare districts, survey years and with the five first principal components, and were analyzed by Snptest 2.5.4-beta3 (Marchini et al. 2007). Any individual of a pair, sharing more than 20% of their genomes, was removed. The genome wide average proportion of alleles shared identical by state (IBS) between any two individuals was determined by plink (Purcell et al. 2007). Chip genotyping and imputation are shown in detail in Online Resource 2.

### Homology search strategy

In order to find the closest *C. elegans* homologs for the ten human genes discovered in the GWAS, four databases were consulted hierarchically. Firstly, the ‘NCBI HomoloGene’ database was checked (ncbi.nlm.nih.gov/homologene; Release 68). It provides an environment for the automated detection of homologs among annotated genes of genomes across multiple well-documented species. Secondly, the ‘Ortholist 2’ database was used, which is compiled by a meta-analysis of six orthology-prediction methods (ortholist.shaye-lab.org) (Kim et al. 2018). Thirdly, the software package for sequence analysis ‘HMMER’ was consulted (release 3.3.2, 2020-11-27). The canonical protein sequence in the UniProt database for each gene in the GWAS study was used as a query in a phmmer search (www.ebi.ac.uk/Tools/hmmer/search/phmmer). The default parameters were used, with the exception to restrict the search to the proteome of *C. elegans*. Only hits with an E-value below 0.01 are reported. Fourthly, ‘Aceview’ (ncbi.nlm.nih.gov/IEB/Research/Acembly/index.html; last update: 2012-10-16) was used, which provides a database of public mRNA sequences. Thirteen homologs found in HomoloGene, Ortholist 2 and both in Aceview and HMMER were selected for further evaluation in the healthspan experiments.

### C. elegans maintenance

The wild-type *C. elegans* strain N2 (Bristol) as well as the strains RW1596, GA410, TJ375, SJ4100, SJ4005 (for details, see Online Resource 3) were obtained from the *Caenorhabditis* Genetics Center (CGC) (Minneapolis, MN, USA). Nematodes were maintained according to Brenner (1974) at 20–22 °C on 96 mm nematode growth medium (NGM) agar plates seeded with the *Escherichia coli* strain HT115 (Ahringer Library, Source BioScience, Nottingham, UK). Synchronized populations were regularly generated by dissolving young adults in a 3% sodium hypochlorite solution until eggs were isolated, based on a protocol from Stiernagle (2006). The obtained eggs hatched in M9 buffer overnight at 20–22 °C and were transferred to new NGM plates the following day.

### RNAi treatment

The different RNAi feeding strains were obtained from the Ahringer Library (Source BioScience, Nottingham, UK). Each strain includes the L4440 vector containing an insert of the respective target gene. The insert of the RNAi clones was verified by sequencing using the pL4440-dest-RNAi universal primers (Forward: GTTTTCCCAGTCACGACGTT; Reverse: TGGATAACCGTATTACCGCC). One colony was isolated and used as a template for the PCR (TopTaq PCR kit Qiagen). Sequencing was performed with the same primers using an ABI3130XL sequencer (Applied Biosystems).

To prepare a fresh liquid bacterial culture, single colonies from LB-plates (containing 2 mg/mL carbenicillin and 10 µg/ml tetracycline) were used to inoculate liquid LB medium (Miller) including 2 mg/ml carbenicillin. After growing for a maximum of 18 hours at 37 °C, 1 mM IPTG was added, and incubation was continued for two additional hours. After washing with 3 g/L NaCl, the strains were concentrated to OD_595_ = 9. The bacteria were stored at 4 °C for no longer than seven days. One to two days before treatment initiation, the bacteria were distributed on NGM agar plates containing 1 mM IPTG and 2 mg/ml carbenicillin. Nematodes in the first larval stage (L1) were fed with the HT115 strain including the L4440 vector without insert (hereinafter called “empty vector” or “EV”). The worms were transferred to the RNAi plates at the larval stage 4 (L4) to avoid any interference with development, and 100 µM 5-fluorodeoxyuridine (FUdR) was added to prevent reproduction. Worms were regularly transferred to fresh RNAi plates to prevent them from running out of food and were fed with the RNAi bacteria throughout their adulthood. The EV strain was used as a control in all experiments.

### Salt chemotaxis assay

CTX agar (10 g agar, 5 mM KH_2_PO_4_/K_2_HPO_4_, 1 mM CaCl_2_ and 1 mM MgSO_4_) was poured into large (92 mm) Petri plates and quartered by labelling on the backside as explained by Margie et al. (2013). In addition, 15 ml CTX agar spiked with 100 mM NaCl was poured into medium-sized (60 mm) plates. Twenty-two hours before test performance, two plugs were cut out of the medium plate by using the wide opening of a 1000 µl pipette tip. Two opposing quadrants were labelled and the plugs were placed on defined positions until test initiation.

The chemotaxis (CTX) assay was performed on the 3rd and 7th day of adulthood. Only cut tips with a wide opening were used to transfer worms in order to avoid stress and injuries. Approximately 150 RNAi-treated individuals were washed from a medium sized NGM plate with CTX buffer (5 mM KH_2_PO_4_/K_2_HPO_4_, 1 mM CaCl_2_ and 1 mM MgSO_4_) and collected in 2 ml tubes. After the worms settled by gravity, the supernatant was removed and replaced by a small amount of fresh CTX buffer. Washing was repeated if bacteria were still detected under the microscope. After removing the plugs and settling of the worms, the nematodes were transferred by using a volume of 80 µl to the centre of a CTX plate. As soon as the liquid was air-dried, 1 µl 1 M NaN_3_ was added to each quadrant to anaesthetize arriving animals. After incubation for 1 hour and 1.5 hour (on the 3rd and 7th day of adulthood, respectively) in the dark, the NaN_3_ treatment was repeated. The plates were directly moved to 4 °C (3rd day of adulthood) or incubated at 22 °C overnight before transferring them to 4 °C (7th day of adulthood). The different handling of the two ageing groups is reasonable given the dissimilar movement speed of the animals. Thereafter, all nematodes in the four quadrants were counted to calculate the chemotaxis index (CI) = (n [NaCl-quadrants] − n [control-quadrants])/n [NaCl-quadrants + control-quadrants]. The number of nematodes left in the starting circle was counted in addition to verify a good performance of the test, but these worms were not included in the calculation. An above-average number of worms at this spot could indicate a high bacterial load or movement-problems due to injuries.

### Muscle integrity analysis

The translational reporter strain RW1596 (see Online Resource 3) was used to evaluate muscle integrity. Adult day 1 (only for the young control on empty L4440) and day 14 old worms were collected and immobilized on a slide with a 2.5% agar pad and 0.05 µm Polybead® Polystyrene beads (Polysciences, USA). Confocal images were acquired with a Nikon TiE-C2 confocal microscope using a 488 nm solid state laser and a 40x CFI Plan Apochromat objective (NA 1.25, water immersion). Individual muscle cells were imaged using a 2x scan magnification. On average, two muscle cells were imaged per worm, and images were taken of at least ten animals per condition. In total, three independent experiments were run for all conditions. Images were processed using the image processing freeware Fiji (Schindelin et al. 2012), and morphological features that significantly changed with age were extracted using a dedicated script that is available at https://github.com/DeVosLab/MuscleMetrics . In brief, the analysis pre-processes the images by local contrast enhancement and background subtraction, after which the myofilaments are selectively enhanced by means of a directional second order derivative and binarized according to Yen’s autothreshold algorithm. Next to the morphological descriptors that are extracted by default in FIJI, the analysis also measures local thickness, cytoskeleton complexity, and curvature variations of individual myofilaments.

### Pathogenic stress resistance assay

*Photorhabdus luminescens* (subspec. Laumondii, strain TT01; obtained from the Leibniz Institute DSMZ-German Collection of Microorganisms and Cell Cultures GmbH, Braunschweig, Germany) was selected to induce pathogenic stress in *C. elegans* according to Hoinville and Wollenberg (2018) and Sato et al. (2014). A frozen glycerol stock of *P. luminescens* bacteria was used to inoculate liquid LB medium (Lennox). After growing at 28 °C with agitation for 48 hours, the bacteria were concentrated to a final OD_595_ = 9 and mixed (1:1) with the respective RNAi or EV strain. 200 µl of this mixture was thoroughly distributed and dried on small (35 mm) NGM agar plates (including 1 mM IPTG but without carbenicillin) and incubated for an additional 48 hours at 28 °C. As described above, the treatment of the nematodes with the respective RNAi or EV strain started at the L4 stage. Thereafter, on the 3rd and 7th day of adulthood, nematodes were transferred to the *P. luminescens*-containing plates, incubated at 22 °C, and survivors were counted daily by observing their movement after a soft touch with a platinum wire under the microscope. Since pharyngeal pumping of *C. elegans* declines dramatically during ageing (Chow et al. 2006), a sufficient intake of pathogens cannot be guaranteed in worms on the 14th day of adulthood. Thus, the assay was only started on the 3rd and 7th day of adulthood. Each stress assay was performed blinded in at least two biological repeats.

### Stress reporter assays

The activation of four different cellular stress pathways involved in lifespan regulation was verified, including the activation of DAF-16 (general stress response), HSF-1 (cytoplasmic proteotoxic stress) and the induction of the mitochondrial and endoplasmic unfolded protein response (mtUPR and erUPR, respectively). An overview of the GFP strains used for the stress reporter assays can be found in Online Resource 3. L1 worms were put on EV plates until L4 stage. Next, worms were transferred to the RNAi plates and FUdR (final concentration 100 µM) was added. To validate the stress reporter assay, positive controls were run in parallel for which corresponding treatments were started from L1 stage. At day 3 of adulthood, worms were collected and washed twice with S-buffer to remove the bacteria. To measure the GFP fluorescence, 100 µl of worm suspension was transferred to a Greiner 96 flat-bottom black polystyrol plate (Sigma-Aldrich) using a glass tip. The GFP signal of two technical replicates was measured with a Perkin Elmer Wallac 1420 Victor^2^ Microplate Reader at an excitation and emission wavelength of 485 nm and 535 nm, respectively. Measurements were normalized to total protein level, estimated by a Pierce^TM^ BCA^TM^ Protein Assay kit (Thermo Scientific).

### Lifespan assay

About 35 L4-stage synchronized nematodes were transferred to a NGM agar plate containing IPTG, carbenicillin and the respective RNAi-bacteria (as explained in 2.4). Surviving and dead worms were counted daily until all worms had died. Five plates were observed per treatment group. Worms that displayed vulval extrusion or desiccation due to crawling off the agar were censored.

### Data analysis

The chemotaxis assay was statistically analysed with R version 4.0.3 using One-Way ANOVA and the Dunnett’s multiple comparison *post hoc* test. The statistical analyses of the stress resistance and lifespan assays were carried out using a log-rank test with subsequent Bonferroni correction via the Online Application for Survival analysis OASIS 2 (Han et al. 2016). The muscle integrity data were processed in RStudio with R version 4.0.0 and the statistical analysis was conducted in GraphPad Prism 9 applying a Kruskal-Wallis test and Dunn’s multiple comparisons test. For the comparison of the age standard, a Mann-Whitney test was applied. Stress reporter data were analysed using One-Way ANOVA and the Dunnett’s multiple comparison *post hoc* test. Graphs were prepared with GraphPad Prism 9.

## Results

### GWAS analyses reveal ten human genes as potential healthspan modulators

To identify genes affecting healthspan, we performed two genome-wide association studies using Finnish FINRISK population surveys from the years 1992, 1997, 2002 and 2007. At first, we compared healthy individuals (n = 750) vs. patients suffering from common age-related disorders (further referred to as unhealthy, n = 1502), who had reached a minimum age of 75 years (GWAS1). In a second comparison (GWAS2), we expanded the latter group with a set of individuals with all-cause mortality under 75 years (n = 2663).

**Fig. 1:**
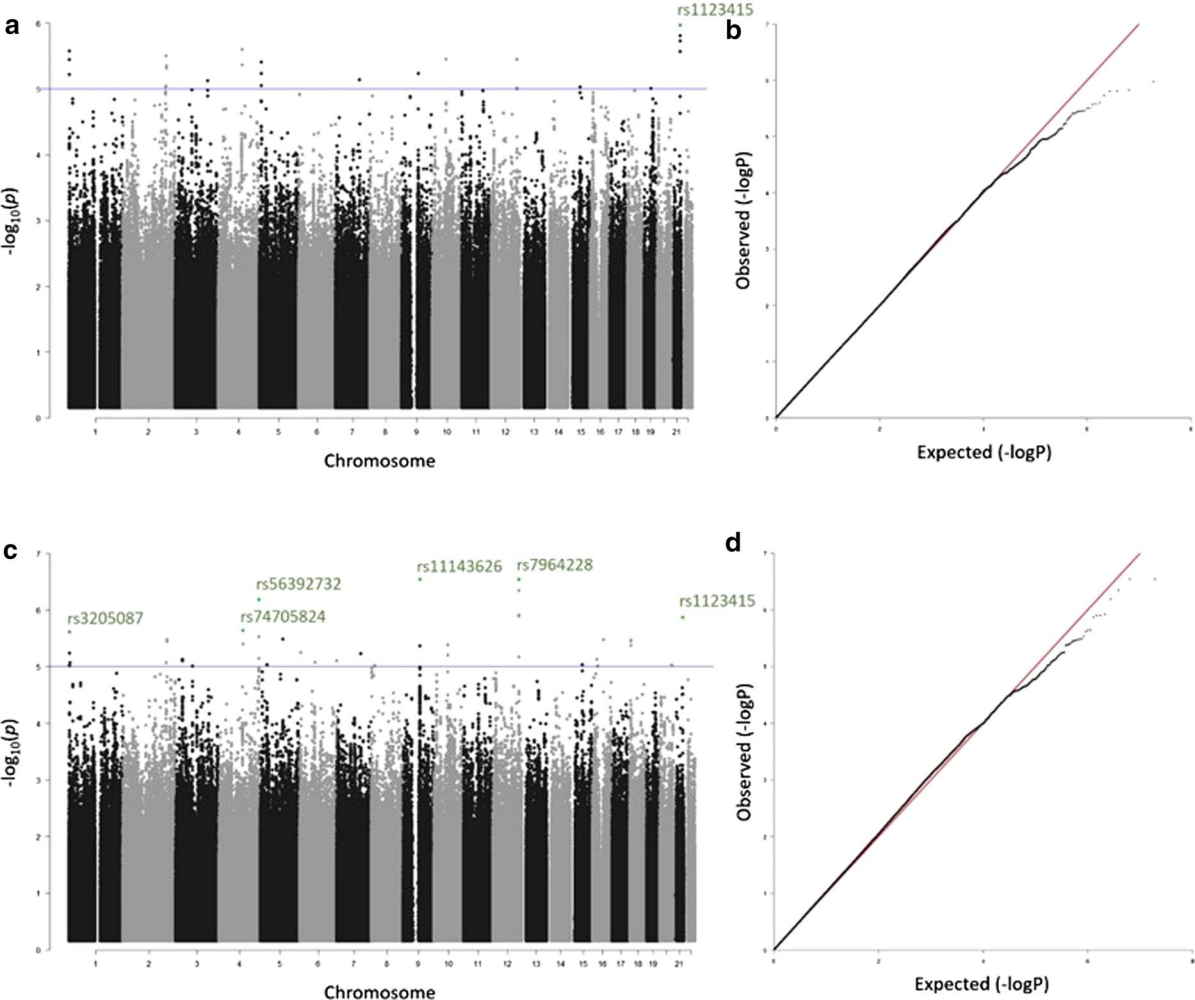
Selected SNPs from two GWASs Manhattan plots **(a**, **c)** and Q-Q plots of observed versus expected-log(P) **(b**, **d)** of the GWAS1 **(a**, **b)** and GWAS2 study (**c**, **d**) of increased healthspan in individuals over the age of 75

We tested ~9.6 million nucleotide variations for association with a healthy lifespan, covering the autosomal part of the human genome (Fig. 1). We identified six human SNPs, based on GWAS2 and also supported by GWAS1 (for details, see Online Resource 4) that differ significantly between the healthy and unhealthy cohort, and thus showed potential for healthspan modulation (Table 1, Fig. 1), with SNPs rs11143626 and rs7964228 having the lowest p-values. The G-allele of rs11143626 (C/G) was shown to have a positive impact on healthy lifespan. The SNP was found in a gene desert on chromosome 9, and the closest gene annexin A1 (*ANXA1*) was identified at a distance of 400 kb. ANXA1 is an inhibitor of phospholipase A2, and has Ca^2+^ and phospholipid binding sites. *ANXA1* is expressed in monocytes/macrophages and neutrophils and its anti-inflammatory action interferes the leukocyte migration and platelet aggregation (Parente and Solito 2004). ANXA1 has been shown to inhibit the development of atherosclerosis via an anti-inflammatory reaction (Fredman et al. 2015). SNP rs7964228 (G/A) locates near the *ACADS* gene on chromosome 12, with the minor A-allele having a negative impact on healthspan. The gene encodes a tetrameric mitochondrial flavoprotein, and belongs to the acyl-CoA dehydrogenase family. The enzyme catalyzes the first step of the mitochondrial fatty acid beta-oxidation pathway. SNP rs56392732 (C/T), (T having a negative impact on healthspan), was ranked third and locates to chromosome 4 in the middle of a gene cluster (*WWC2*-*CLDN22/24*-*CDKN2AIP*). The *WWC2* gene encodes a member of the WW-and-C2-domain-containing family of proteins and modulates hippo pathway signaling (Wennmann et al. 2014). *CLDN22* and *CLDN24* genes encode members of the claudin family. Claudins are integral membrane proteins and components of tight junction strands. The *CDKN2AIP* gene encodes a CDKN2A-interacting protein, which regulates the DNA damage response through several different signaling pathways. CDKN2AIP interacts with MDM2, p16 and p53 and is a regulator of the p16, p53/p21 pathways (Wadhwa et al. 2017). The next SNP rs1123415 (G/A) is located between the genes *UBASH3A* and *RSPH1*, with the A-allele having a positive effect on healthy lifespan. The ubiquitin-associated and SH3-domain-containing A (UBASH3A) protein negatively regulates T-cell signalling (Ge et al. 2019). *RSPH1* encodes a male meiotic metaphase chromosome-associated acidic protein. This gene is expressed in tissues with motile cilia or flagella, such as the lungs, trachea and testes. Mutations in this gene result in primary ciliary dyskinesia (Onoufriadis et al. 2014). SNP rs74705824 (C/G) near the *ELOVL6* gene was observed to have the fifth-lowest p-value, and the G-allele had a positive impact on healthspan. ELOVL6 catalyses the first and rate-limiting reaction of the four reactions that constitute the long-chain fatty acids elongation cycle. Elongation and desaturation are crucial steps in the *de novo* synthesis of long‐chain fatty acids that define their function and metabolic destiny (Sunaga et al. 2016). Interestingly, mice deficient in Elovl6 (Elovl6−/−) are protected against diet‐induced insulin resistance despite their hepatosteatosis and obesity being comparable to that in wild‐type mice (Matsuzaka et al. 2007). Finally, we detected rs3205087 (A/G), the G-allele having a positive effect on healthspan, in the DNA fragmentation factor (*DFFB*) gene located on the short arm of chromosome 1p36. DFFB is a nuclease that induces DNA fragmentation and chromatin condensation during apoptosis; it degrades naked DNA and induces apoptotic morphology. It forms a heterodimer with DFFA, the substrate for caspase-3 and triggers DNA fragmentation during apoptosis (Han et al. 2020).

**Table 1:** Discovered SNPs with linked human genes

SNP locus	Human gene	Location of the gene	Physical distance of the SNP to the gene
rs3205087	*DFFB*	1p36.32	Exonic region of *DFFB*
rs74705824	*ELOVL6*	4q25	35.2 kb
rs56392732	*WWC2*	4q35.1	94.2 kb
	*CDKN2AIP*	4q35.1	29.7 kb
	*CLDN22*	4q35.1	94.7 kb
	*CLDN24*	4q35.1	92.5 kb
rs11143626	*ANXA1*	9q21.13	416.8 kb
rs7964228	*ACADS*	12q24.31	1.5 kb
rs1123415	*UBASH3A*	21q22.3	7.5 kb
	*RSPH1*	21q22.3	17.3 kb

### Identification of putative healthspan-related genes in C. elegans

*C. elegans* homologs for the ten human genes were sought in four different databases: NCBI HomoloGene, Ortholist 2, HMMER and Aceview. Hits found on the NCBI HomoloGene database and Ortholist 2 database resulted in a high-confidence list of potential *C. elegan*s homologs. Combined positive hits from both HMMER and Aceview were also included in our final list of selected candidate genes, which are highlighted with a grey background and bold letters (Online Resource 5). For *WWC2*, three potential *C. elegans* homologs (*wwp-1*, *frm-8* and *yap-1*) were selected based on conserved domain homology.

In the case of *ACADS*, we decided to include the genes which were also found in the HMMER and Aceview search, in addition to the Ortholist 2 genes. Although *acdh-1* and *acdh-3* are the closest sequence homologues to *ACADS* (Acyl-CoA dehydrogenase, C-2 to C-3 short chain), they are not true *ACADS* orthologues. Sequence-wise, they more closely resemble *ACADSB* (encoding short/branched chain specific acyl-CoA dehydrogenase) (Watson et al. 2013). *C. elegans* has lost *ACADS*, but contains multiple copies of *ACADSB* (*achd-1* and *acdh-3*) and *ACADM* (acyl-Coenzyme A dehydrogenase, C-4 – C-12 straight chain; *acdh-7*, *achd-8* and *acdh-10*) which all are functional (Swigonová et al. 2009). These genes may have taken over some of the functions of *ACADS*, making it worthwhile to include them in our screen. For the same reason *ivd-1* was included. In total, we selected 13 *C. elegans* homologs for further evaluation in healthspan experiments.

### Downregulation of wwp-1 and yap-1 modulate chemotactic behaviour

**Fig. 2:**
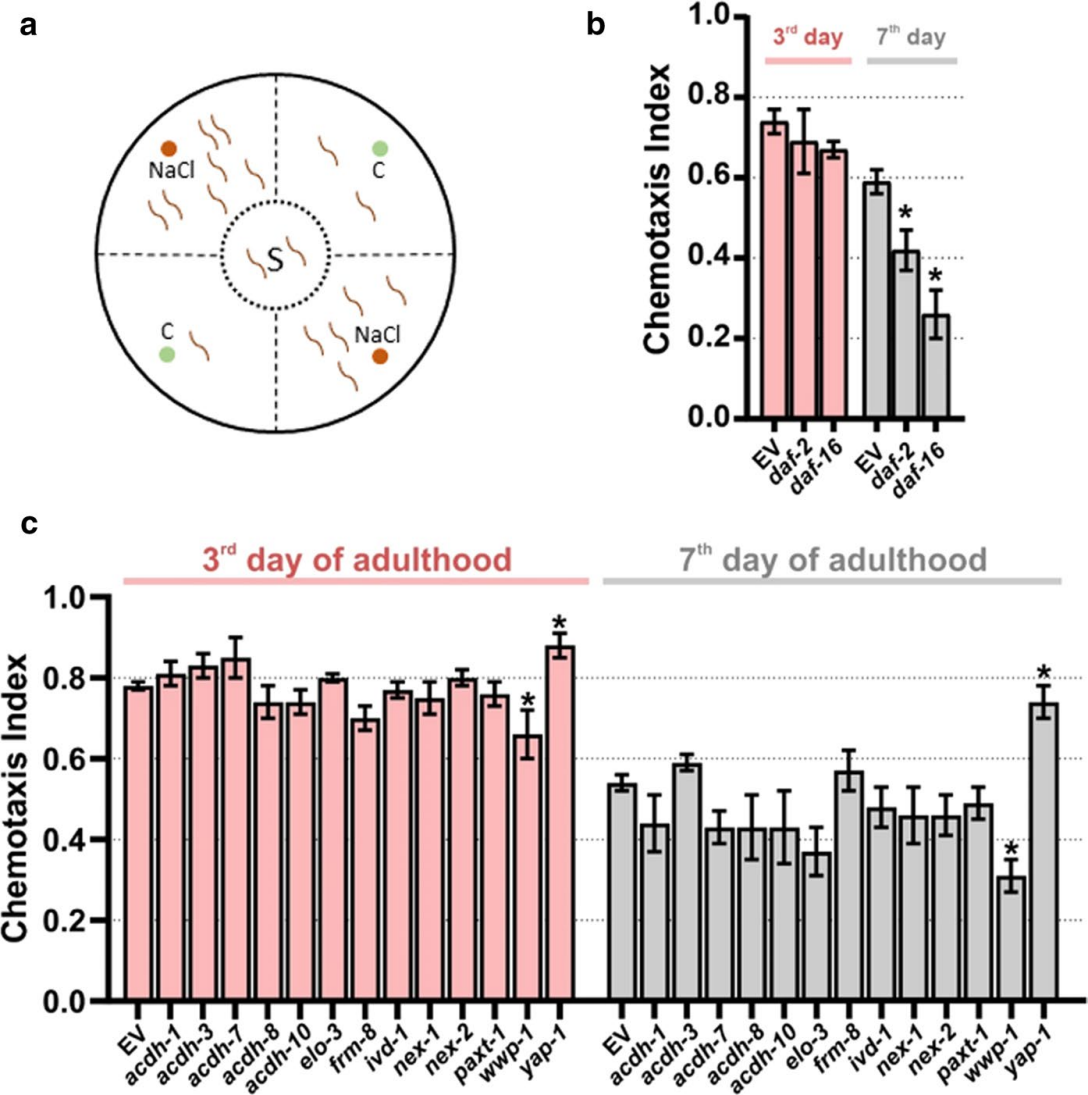
Chemotactic capacities after RNAi treatment. The chemotaxis-plates were divided into two NaCl-quadrants, two control-quadrants ‘C’ and the starting circle of the nematodes ‘S’ (**a**). The four small spots are equally distributed for positioning the NaCl plugs and the NaN_3_ application. The attraction towards NaCl was measured in RNAi-treated and control (EV) nematodes. Each bar represents three independent biological repeats with n ≥ 443 and n ≥ 619 (**b**) and n ≥ 852 and n ≥ 732 (**c**) wild type nematodes on the 3rd and 7th day of adulthood, respectively. The error bars display SEM. Significant differences were determined with *p < 0.05 by One-Way ANOVA and the Dunnett’s multiple comparison *post hoc* test

Sensory perception decreases with age in humans (Nordin 2017; Somekawa et al. 2017) as well as in *C. elegans* (Collins et al. 2008). More specifically, the worm’s activity of the sensory neurons responsible for detecting chemical stimuli declines with age (Leinwand et al. 2015). To detect changes in the chemotactic behaviour, we exploited the fact that *C. elegans* links the environmental salt concentration during its cultivation to the presence of food. As a result, the worm navigates along the salt gradient on the prepared chemotaxis plates in search of the desired food. To exclude the possibility that different locomotion-abilities between the treatment groups changed the chemotaxis index, the plate design was selected according to Margie et al. (2013) (Fig. 2a). Nematodes which didn’t move out of the starting circle were censored. The mobile worms had to make a choice between the NaCl-spiked and control quadrants. Due to the anaesthetic application, the first decision was also the final one. Nematodes were tested at young (3rd day of adulthood) and medium (7th day of adulthood) age. Older animals (10th day of adulthood and older) showed severe movement disabilities, and were thus not suitable for this assay.

The sensory impairment during ageing was verified in the chemotaxis assay performed in this study (Fig. 2b and 2c). The chemotaxis index (CI) in the control group (EV) declined from 0.75–0.78 (3rd day of adulthood) to 0.54–0.59 (7th day of adulthood), whereas a CI of  + 1.0 corresponds to maximal attraction and − 1.0 to maximal repulsion.

To evaluate through RNAi-mediated gene silencing of ageing-related genes whether the assay detects modifications of the sensory perception, the *daf-2* and *daf-16* knockdown groups were used as internal controls. Both genes are components of the insulin/IGF-1 pathway, which is a key player in the genetics of ageing (Kenyon 2010) and is involved in salt chemotaxis learning in *C. elegans* (Tomioka et al. 2006). Interestingly, both, *daf-2* and *daf-16* knockout strains feature an impaired salt chemotaxis learning (Vellai et al. 2006; Tomioka et al. 2006). In our chemotaxis assay, no significant differences could be determined for *daf-2*- and *daf-16*-RNAi-treated worms in comparison to the control on the 3rd day of adulthood. However, on the 7th day of adulthood both RNAi treatments resulted in a significant decrease of the CIs, and *daf-16*-RNAi-treated worms performed worse than *daf-2* knockdowns (Fig. 2b). Thus, the assay revealed an age-related decrease in salt chemoattraction of *daf-2* and *daf-16* knockdowns compared to an EV control.

The general age-dependence of chemotactic capacities was visible in all tested RNAi-groups. However, this decline seemed to be delayed in nematodes after downregulation of *yap-1* (Fig. 2c), which led to an improved maintenance of chemotactic abilities over time. Compared to the control, *yap-1*-RNAi-treated worms have a chemotaxis index that is 13% higher on the 3rd day and 37% higher on the 7th day of adulthood. This clearly indicates a negative role of YAP-1 for age-dependent sensory perception. In contrast, the downregulation of *wwp-1* resulted in the deterioration of salt chemotaxis in both age groups, with a reduction of about 20% (CI = 0.66 ± 0.06) on the 3rd day and 70% (CI = 0.31 ± 0.04) on the 7th day of adulthood, respectively, compared to the EV control. This accelerated decline of the CI indicates healthspan shortening. Thus, *yap-1* and *wwp-1* modulate age-related chemotaxis decline in opposite directions.

### Improvement of muscle integrity by downregulating paxt-1 and acdh-3

**Fig. 3:**
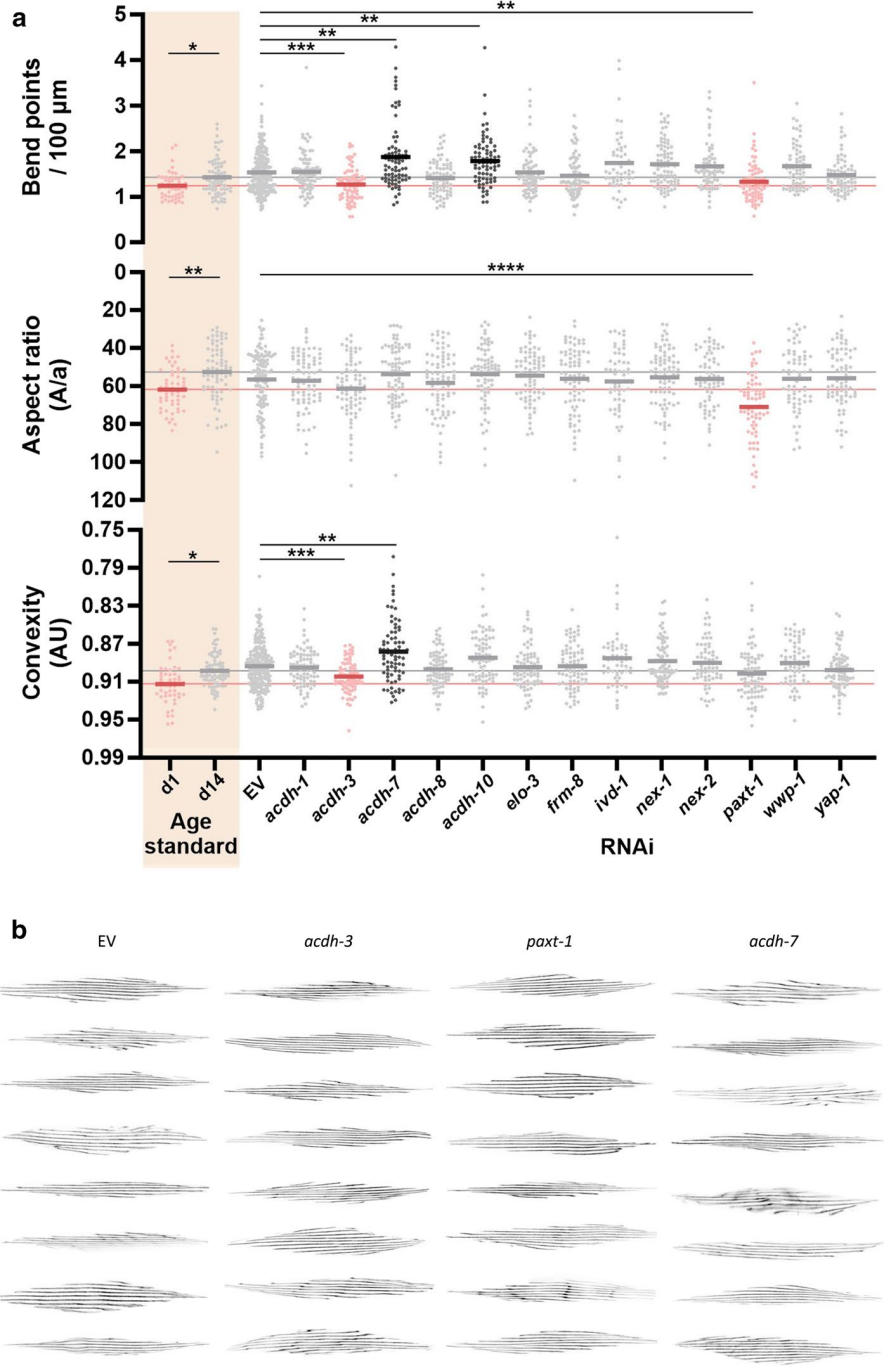
Muscle integrity after RNAi treatments. **a** Three object features are extracted to evaluate muscle integrity at advanced age in MYO-3::GFP worms. Age standard indicates the expected changes for these features in old (day 14—light grey) versus young worms (day 1—pink). RNAi-treatment targeting the GWAS-based candidates was performed until day 14 of adulthood. RNAi-conditions in pink indicate a significant shift towards the phenotype observed in young worms; dark grey indicates conditions with a more severe phenotype compared to old EV-treated day-14 worms. Significant differences were determined by Kruskal-Wallis test and Dunn’s Multiple comparisons test, and marked with *p < 0.05, **p < 0.01, ***p < 0.001, and ****p < 0.0001. For the comparison of the age standard, a Mann-Whitney test was applied. **b** The strain RW1596 expresses GFP-tagged myosin heavy chain A in the body wall muscle. Shown are random example confocal images of muscles in EV, *acdh-3*, *paxt-1*, and *acdh-7* RNAi treated transgenic nematodes at day 14

A key component of human late-life frailty is sarcopenia, a phenomenon which has also been observed in old *C. elegans* worms. At the cellular level, myofilament organization tends to deviate from the tight parallel orientation seen in young worms (Christian and Benian 2020; Herndon et al. 2002). Hence, the delayed onset of sarcopenia can be considered a proxy of extended healthspan. In order to evaluate worm muscle integrity, we used a transgenic MYO-3::GFP strain expressing GFP-tagged myosin heavy chain (MHC) A in the body wall muscle. Loss of muscle integrity in old worms can be inferred from muscle filament structure analysis (Dhondt et al. 2021). The increasing distortion and fragmentation of otherwise well-aligned myofilaments is reflected by several parameters in old worms (Fig. 3a). For instance, myofilaments in old worms tend to deviate significantly from the tight convex shape observed in young control animals, and the average number of bend points per 100 µm increases significantly. These two parameters clearly indicate increasing filament curvature. In addition, the aspect ratio significantly drops in old day 14 animals, corresponding to a higher degree of fragmentation. To evaluate the effect of the healthspan-related candidate genes on muscle integrity, we compared the same myofilament descriptors in RNAi-treated animals versus EV-treated controls on day 14 of adulthood **(**Fig. 3a**)**.

RNAi targeting of *acdh-3* and *paxt-1* resulted in a significantly lower number of bend points. In addition, *acdh-3* knockdown seems to maintain the tight shape (or convexity) observed in young controls. Knockdown of *paxt-1* led to decreased fragmentation of myofilaments, for which the aspect ratio is significantly higher compared to EV-treated controls. On the other hand, we observed that *acdh-7* and *acdh-10* RNAi treatment caused a more severe phenotype compared with the day-14 control worms, for which we observed significantly more bend points. Additionally, reduced *acdh-7* expression seems to result in a more serious loss of shape represented by the convexity parameter. Random example images for nematodes treated with control and RNAi (*acdh-3, acdh-7, and paxt-1*) bacteria are shown in Figure 3b and the full set of images is available in Online Resource 11. Overall, knockdown of *acdh-3* and *paxt-1* resulted in the most beneficial effect.

### Numerous genes are involved in pathogenic stress resistance of moderate aged nematodes

Stress resistance is a key feature for the maintenance of healthspan and is declining with age in *C. elegans* as well as in humans (Chen et al. 2020; Everman and Morgan 2018; Kourtis and Tavernarakis 2011). For humans, the resistance against pathogens is a major concern in elderly (Gavazzi and Krause 2002; Stavropoulou and Bezirtzoglou 2019). Thus, we focused on pathogenic stress resistance in the nematode model.

**Fig. 4:**
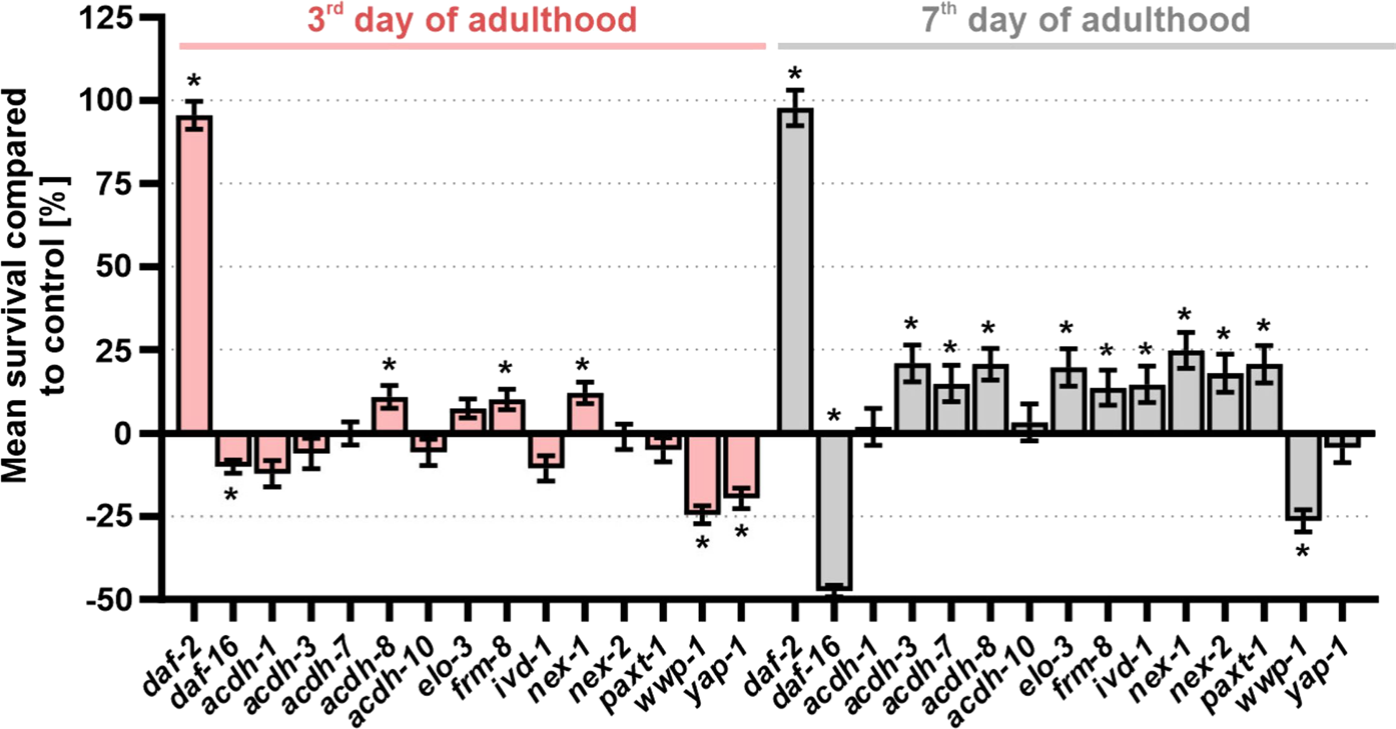
Effect of RNAi-treatments on pathogen resistance. On the 3rd and 7th day of adulthood, RNAi-treated nematodes were transferred to *Photorhabdus luminescens*-covered plates, and the survival was monitored daily. At least two biological repeats were performed, except for *daf-2* and *daf-16* RNAi-treated nematodes, which were only tested once. Significant differences between the survival distributions were determined by log-rank test with subsequent Bonferroni correction, and indicated with *p < 0.05. The mean survival and SEM are shown during pathogen exposure compared to control. Furthermore, the survival curves for the single treatments are shown in Figs. S1 and S2 (Online Resource 8)

Because the insulin/IGF-1 signalling pathway is associated with host defence against *P. luminescens* in *C. elegans* (Garsin et al. 2003), the pathogen stress assay was verified with *daf-2*- and *daf-16*-knocked-down test groups (Fig. 4 and Online Resource 7). The downregulation of *daf-2* led to a pronounced increase in mean survival. The worms lived 81.4% and 64.6% longer when the assay started on the 3rd or 7th day of adulthood, respectively. In contrast, *daf-16*-knocked-down animals had a 7.1% and 40.4% shorter mean survival in the younger or older age class, respectively, when infected with *P. luminescens*. Thus, the applied assay clearly reflects the expected involvement of *daf-2* and *daf-16* in stress resistance.

Despite the presumed reduced pathogen intake on the 7th (compared to the 3rd) day of adulthood (Chow et al. 2006), the decline of pathogen resistance against *P. luminescens* during ageing was visible. The mean survival of the control (EV) during pathogenic stress was 5.77 and 4.63 days when starting the exposure on the 3rd and 7th day of adulthood, respectively (Online Resource 6). When pathogen exposure started on the 3rd day of adulthood, the RNAi treatments targeting *acdh-8*, *frm-8* and *nex-1* resulted in an improved survival, whereas reduced survival was observed with the downregulation of *wwp-1* and *yap-1* (Fig. 4, Online Resource 8 (Fig. S1) and Online Resource 6). For older populations, most RNAi treatments provoked an increase in pathogenic stress resistance (Fig. 4, Online Resource 8 (Fig. S2) and Online Resource 6), whereas only the downregulation of *wwp-1* decreased pathogen resistance. Downregulation of *acdh-1* and *acdh-10* did not provoke any significant change in the survival of both age classes.

Two patterns were dominant when comparing the stress resistance after RNAi-treatments on the 3rd and 7th day of adulthood: a) unchanged resistance in young and improved resistance in older worms (this holds true for *acdh-3*, *acdh-7*, *elo-3*, *ivd-1*, *nex-2*, and *paxt-1*) or b) improved resistance in both age classes with a slightly stronger effect in older worms (this holds true for *acdh-8*, *frm-8*, and *nex-1*). The exceptions for these patterns are found for the downregulation of *yap-1* (decreased survival in young and unchanged survival in older nematodes), and *wwp-1* (decreased survival in both age groups) (Fig. 4, Online Resource 8 and Online Resource 6). Overall, most of the *C. elegans* genes identified based on the GWAS data showed an involvement in pathogen resistance in an age-dependent manner.

### Longevity-related signalling pathways and cellular processes are not activated upon knockdown of GWAS-based genes

**Fig. 5:**
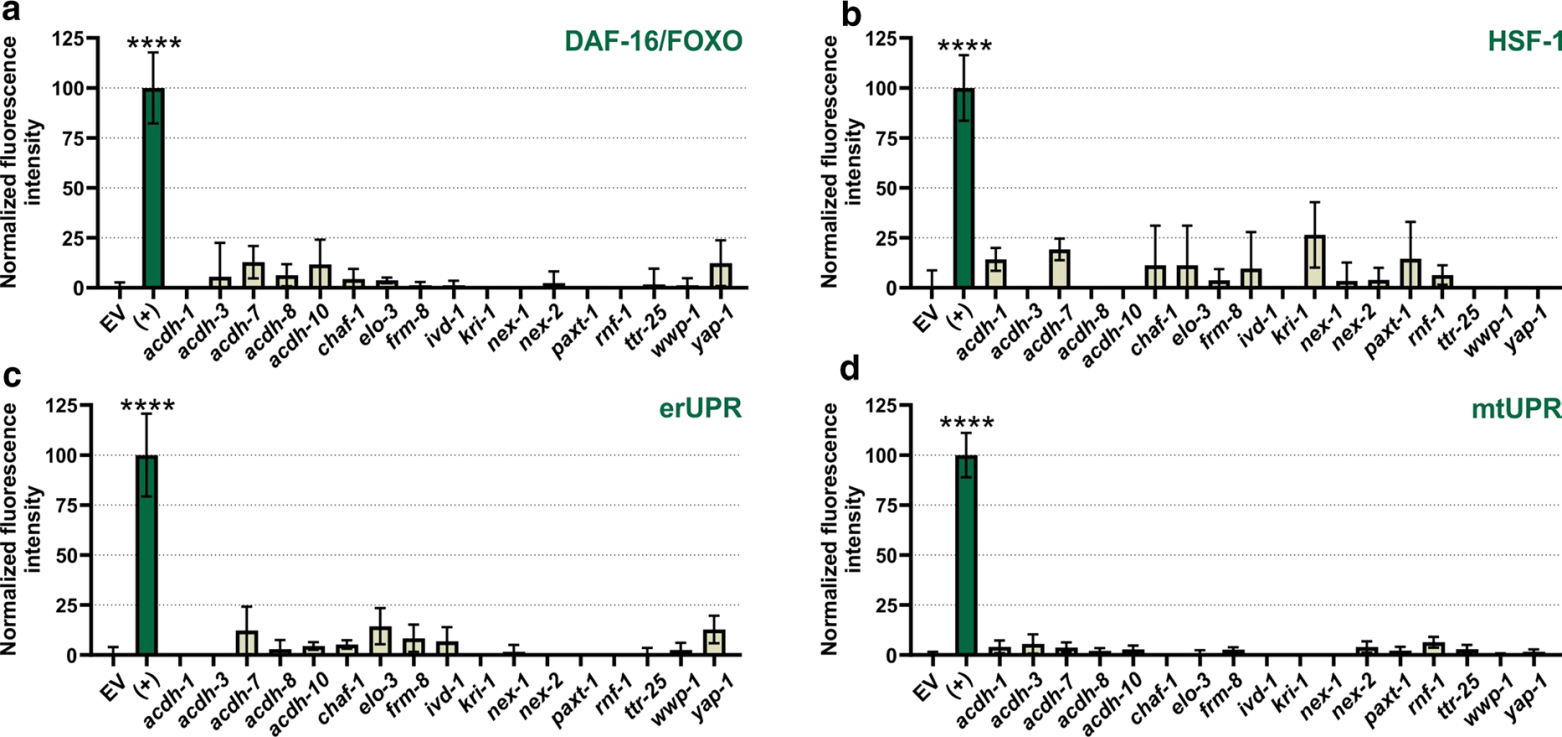
Effect of RNAi-treatments on signaling pathways. Longevity-related signaling pathways and cellular processes are not activated upon knockdown of GWAS genes. (+) indicates the positive controls **a** age-1 (RNAi), **b** heat shock (1h 37°C, 2h recovery at 20°C), **c** mdt-15 (RNAi), and **d** nuo-2 (RNAi). The error bars display SEM. Significant differences were determined by One-Way ANOVA and the Dunnett’s multiple comparison post hoc test and indicated with ****p < 0.0001

Several transcriptional regulators and proteostatic pathways are known to play key roles in lifespan extension in *C. elegans* (Denzel et al. 2019). The conserved FOXO transcription factor DAF-16, for example, modulates stress-related homeostasis and is involved in the regulation of lifespan extension in response to several stimuli (Kenyon 2010). Another regulator of lifespan is the heat shock transcription factor HSF-1 (HSF1), which regulates the expression of chaperones in response to proteostatic stress (Li et al. 2017). Next to these longevity-associated transcription factors, several cellular stress response pathways are known to mediate lifespan extension too, including the unfolded protein response (UPR) in the mitochondria (UPR^mt^) and endoplasmic reticulum (UPR^ER^) (Denzel et al. 2019). We evaluated the activation of these well-studied lifespan mediators using genetic reporter strains, in which GFP is induced upon activation of a specific signalling pathway. To determine whether our GWAS genes activate DAF-16, HSF-1, UPR^mt^ or UPR^ER^, we looked at the activation of their respective targets: SOD-3, HSP-16.2, HSP-6 and HSP-4. None of the RNAi-treatments evoked a significant increase of fluorescence in the reporter worms, which indicates that the genes studied here do not act via DAF-16, HSF-1 or the UPR in mitochondria or endoplasmic reticulum (Fig. 5).

### Knockdown of acdh-7, acdh-8, elo-3, wwp-1, and yap-1 affect lifespan

**Fig. 6:**
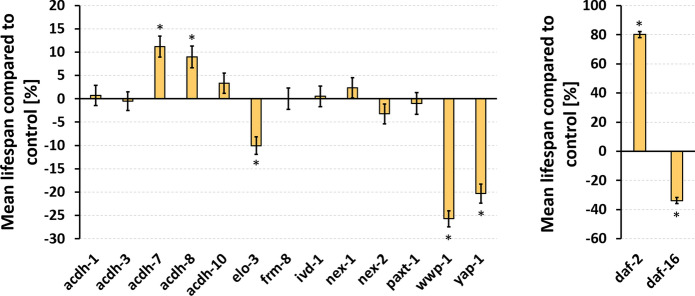
Impact of RNAi-treatments on lifespan. The lifespan of 151–170 RNAi-treated nematodes per treatment group was determined by daily counting of dead and alive worms. The percentage changes of the mean lifespan ± SEM are shown compared to the EV-control. Significant differences were determined by log-rank test with subsequent Bonferroni correction, and indicated with * p < 0.05. The survival curves for the single treatments are shown in Fig. S3 (Online Resource 8).

Since life- and healthspan are not necessarily correlated (Hansen and Kennedy 2016; Saul et al. 2021), the lifespan during RNAi-mediated knockdown of the target genes was determined in addition. The knockdown of *daf-2* and *daf-16* served as internal control, which led to severe changes of the mean lifespan. Nematodes treated with *daf-2* RNAi lived about 80% longer, whereby the mean lifespan of the *daf-16* knockdown group was about 34% shorter than that of the control (Fig. 6). The decrease of the mean lifespan in the *wwp-1* (− 26%) and *yap-1* (− 20%) knockdown groups almost reached the extent of the *daf-16* knockdown effect. Interestingly, the downregulation of *elo-3* also led to a shortened mean lifespan (− 10%). In contrast, RNAi targeting of *acdh-7* and *acdh-8* increased the mean lifespan by 11% and 9%, respectively. Details for the lifespan assay can be found in Online Resource 9 and the survival curves in Online Resource 8 (Fig. S3).

Finally, to verify the efficient downregulation of the target genes *via* RNAi, RNAi-treated and untreated nematodes were collected at the 3rd day of adulthood. By using a modified “single worm qPCR” protocol based on a method from Ly et al. (2015), the expression levels of the target genes were determined (methodological details are presented in Online Resource 10). A significant downregulation (compared to the EV-control) of the targeted transcripts *via* RNAi could be verified in almost all samples. However, the downregulation of *nex-1* did not reach the significance threshold and *nex-2* did not show any knockdown (Online Resource 10). Since the location of the designed primer pairs can have a great influence on the outcome of the RNAi-verification (Shepard et al. 2005), it cannot be excluded that the effect of RNAi is visible by using different primer pairs. This assumption is underlined by the fact that all RNAi bacteria strains were verified by sequencing and that the used RNAi feeding protocol led to the desired downregulation in most RNAi treatment groups.

## Discussion

### Decelerated immunosenescence as a common feature of prolonged healthspan?

Interestingly, the majority of gene knockdowns showed an increase in pathogen resistance of moderately aged nematodes (Table 2). A reason for that might be the initial selection of healthy vs unhealthy cohorts in the GWAS study. The healthy participants were defined as over 75 year-old and free of certain diagnoses: diabetes, cancer, dementia, cardiovascular diseases, COPD, asthma, Crohn’s disease, malabsorption, rheumatic disease or kidney failure. Interestingly, immunosenescence is held (partly) responsible for almost all of these diseases (Fülöp et al. 2016). Immunosenescence is accompanied by inflammageing, which is an age-dependent chronic, low-grade and systemic inflammatory condition (Thomas et al. 2020). Diabetes (Kolb and Mandrup-Poulsen 2010), cancer (Bottazzi et al. 2018), COPD (Cho et al. 2019), cardiovascular diseases (Del Pinto and Ferri 2018; Yu et al. 2016), asthma (Murray and Chotirmall 2015), Crohn’s disease (Li and Shi 2018), rheumatic diseases (Hohensinner et al. 2014) or kidney failure (Sato and Yanagita 2019) are all associated with inflammation and immunosenescence. However, the causal connection between dementia and inflammation is controversial. Wang et al. (2020c) and Cunningham and Hennessy (2015), for example, found evidence for a causal relationship for inflammation in the occurrence of dementia, whereas Tsui and Davis (2018) and Enciu and Popescu (2013) did not. Thus, it is plausible that decelerated immunosenescence could be a common feature of the healthy participants in the GWAS study. Consequently, the majority of genes and their *C. elegans* homologs detected in this study could play a role in immune defence, and thus could explain the wealth of positive results of the pathogen resistance assay (notwithstanding profound differences in the immune system of nematodes and humans).

**Table 2:** Summary of health-related effects of RNAi-treatments in *C. elegans*

Selected *C. elegans* genes	Human homologues gene	Chemotaxis	Chemotaxis	Muscle bend points	Muscle aspect ratio	Muscle convexity	Pathogen resistance	Pathogen resistance
		day 3	day 7	day 14	day 14	day 14	day 3	day 7
*acdh-1*	*ACADS*	NS	NS	NS	NS	NS	NS	NS
*acdh-3*	*ACADS*	NS	NS	improved^a^	NS	improved^a^	NS	improved^a^
*acdh-7*	*ACADS*	NS	NS	impaired^b^	NS	impaired^b^	NS	improved^a^
*acdh-8*	*ACADS*	NS	NS	NS	NS	NS	improved^a^	improved^a^
*acdh-10*	*ACADS*	NS	NS	impaired^b^	NS	NS	NS	NS
*elo-3*	*ELOVL6*	NS	NS	NS	NS	NS	NS	improved^a^
*frm-8*	*WWC2*	NS	NS	NS	NS	NS	improved^a^	improved^a^
*ivd-1*	*ACADS*	NS	NS	NS	NS	NS	NS	improved^a^
*nex-1*	*ANXA1*	NS	NS	NS	NS	NS	improved^a^	improved^a^
*nex-2*	*ANXA1*	NS	NS	NS	NS	NS	NS	improved^a^
*paxt-1*	*CDKN2AIP*	NS	NS	improved^a^	improved^a^	NS	NS	improved^a^
*wwp-1*	*WWC2*	impaired^b^	impaired^b^	NS	NS	NS	impaired^b^	impaired^b^
*yap-1*	*WWC2*	improved^a^	improved^a^	NS	NS	NS	impaired^b^	NS

*NS* not significant

^a^Indicate an improvement of the phenotype,

^b^Indicate a worsening of the phenotype.

Indeed, four of the five human genes selected for the *C. elegans* assays are already implicated in immunosenescence and inflammation. ELOVL6 is important to prevent damage-induced skin inflammation (Nakamura et al. 2018). The hippo signalling pathway, with WWC2 as one of its key components (Wennmann et al. 2014), features critical functions in the innate immune responses against pathogens and in autoimmune diseases (Zhang et al. 2018). CDKN2AIP suppresses basal NF-κB activity (Li et al. 2011), whereby NF-κΒ is a key element in inflammatory signalling and immunosenescence (Bektas et al. 2017; Jose et al. 2017). Furthermore, CDKN2AIP controls the activity and stability of the ribonuclease XRN2, which is associated with the NF-κΒ-repressing factor (Alexandrova et al. 2020). The anti-inflammatory protein ANXA1 is mainly found in macrophages and neutrophils and is associated with inflammatory processes, immune infiltrates in cancer cells (Liang and Li 2021) and immune response during fungal infections (Sanches et al. 2021). Given the anti-inflammatory role of ANXA1, it is surprising that the downregulation of the ANXA1 worm homologs *nex-1* and *nex-2* led to increased survival during pathogenic stress, which suggests distinct roles for ANXA1 and NEX-1/NEX-2.

### yap-1, wwp-1, paxt-1 and several acdh-genes appear to affect distinct healthspan parameters

Arguably the most interesting *C. elegans* genes in this study are *yap-1*, *wwp-1*, *paxt-1* and several *acdh* genes.

The downregulation of *wwp-1* led to severe decline in the performance during the pathogenic stress assay—as well as chemotaxis assay. Surprisingly, muscle quality was not impaired. Chen et al. (2010) already showed the importance of the WW domain protein WWP-1 in pathogenic defence and its connection to the insulin-like signalling cascade in *C. elegans*. Furthermore, *wwp-1* is essential for lifespan extension caused by different dietary restriction (DR) regimes. It is assumed that WWP-1 acts in concert with the ubiquitin conjugating enzyme UBC-18, activated by environmental stress or DR, which leads to stress resistance and longevity (Carrano and Hunter 2015; Carrano et al. 2009). In concert with XPA-1, WWP-1 is also involved in DNA repair to cope UV irradiation stress (Astin et al. 2008).

The transcriptional co-activator YAP-1, which is part of the conserved Hippo pathway (Lee et al. 2019), also appears not to play a role in maintaining muscle integrity; however, RNAi targeting of *yap-1* led to improvements in sensory perception in young and moderately aged worms. The pathogen vulnerability, however, is increased in young worms and unchanged in moderately aged nematodes. This could be further evidence for the function of YAP-1 as an antagonistic pleiotropy factor (Iwasa et al. 2013). Besides the hypodermis (Spencer et al. 2011) and muscle cells (Ma et al. 2016), *yap-1* is mainly expressed in sensory neurons (Smith et al. 2010), which fits well with the discovered involvement of YAP-1 in sensory perception. Moreover, YAP-1 is involved in the Wnt-mediated polarization of neurons during development (Lee et al. 2018). Recently, (Ma et al. 2020) found evidence that YAP-1 is activated by the pathogen induced disruption of the intestinal epithelial barrier independently of the Hippo pathway. Furthermore, they showed that *yap-1* mutants are hypersensitive to *P. aeruginosa* and *S. Typhimurium* infections in early adulthood, which parallels our results in the *P. luminescens* assay.

Both, *wwp-1* and *yap-1*, were selected in this study due to their homologies to WWC2. In humans, WWC2 was found to be a positive regulator of the Hippo pathway, which is responsible for cell differentiation, proliferation and apoptosis and plays a crucial role in tumour formation (Höffken et al. 2021). To name only a few studies, the level of WWC2 is low in pancreatic cancer (Wang et al. 2020a) and lung cancer (Wang et al. 2020b) cells and low abundance of WWC2 is associated with a poor prognosis in hepatocellular carcinoma (Zhang et al. 2017). No data about the involvement of WWC2 in general health maintenance during ageing are published so far. However, due to the GWAS results and since two of its homologs showed involvement in sensory perception and pathogenic defence in *C. elegans*, this protein deserves a closer look regarding its relevance in ageing.

The downregulation of *paxt-1* led to improved muscle integrity on the 14th day of adulthood and increased pathogen resistance on the 7th day of adulthood. It was previously shown that the depletion of *paxt-1* in *C. elegans* mutants results in reduced XRN-2 levels with severe consequences for larval survival (Miki et al. 2014). Zugasti et al. (2016) demonstrated that RNAi targeting *xrn-2* starting at L1 leads to reduced expression of the antimicrobial peptide *nlp-29* in young adults. However, these results are not necessarily comparable, since we chose to start the RNAi treatment during the last larval stage (L4). This ensures an unaffected body development and concentrates the effects of RNAi on the adult nematode during the ageing process. Furthermore, to improve visibility of healthspan effects of the RNAi treatments, we used aged nematodes for behavioural, ultrastructural and molecular phenotyping. Nevertheless, the enhanced pathogen defence and muscle integrity after *paxt-1* knockdown observed in our study is rather surprising.

Here, the gene *paxt-1* was selected due to its homology to the CDKN2A interacting protein CDKN2AIP, also known as CARF (Collaborator of ARF). *CDKN2AIP* expression level changes in response to stress in fibroblasts, whereas the type of stress determines, whether the transcription is up- or downregulated (Kalra et al. 2020). It was shown that CDKN2AIP is a regulator of the p16, p53/p21 pathways (Wadhwa et al. 2017) and regulates cell proliferation fate in a dose-dependent manner: Slight overexpression leads to growth arrest and senescence, strong overexpression to pro-proliferation and malignant transformation, whereas knockdown induces apoptosis (Wadhwa et al. 2017). Thus, it is not surprising that CDKN2AIP represents a potential therapeutic target for cancer treatments (Kalra et al. 2018) and is suggested to be an important factor in the ageing process (Cheung et al. 2010).

The knockdown of the *acdh*-genes resulted in a very heterogeneous response. RNAi targeting of *acdh-7* and *acdh-10* was detrimental for muscle integrity, but the knockdown of *acdh-7* additionally led to an improved pathogen resistance. Knockdown of *acdh-3* improved muscle quality and resulted in longer survival under pathogenic stress conditions. Reducing *acdh-8* abundance was solely beneficial for pathogenic resistance. Only the knockdown of *acdh-1* had no effect on any of the tested healthspan parameters. Our results mirror the heterogenicity of these acyl CoA dehydrogenase genes in other reports. While no phenotype descriptions were found for the downregulation of *acdh-7*, knockdown of *acdh-3* leads to decreased peptide uptake in intestinal cells (Benner et al. 2011), downregulation of *acdh-8* to reduced fat content (Ashrafi et al. 2003), and *acdh-10* knockdown to increased fat content in serotonin treated nematodes (Srinivasan et al. 2008). Interestingly, the knockout of *acdh-1* leads to hypersensitivity towards propionic acid (Watson et al. 2014) and reduced lifespan (Radeke and Herman 2020) and the knockdown *via* RNAi to reduced lifespan in the dietary restriction mutant *eat-2* (Yuan et al. 2012). Based on that, it is surprising that we could not find phenotypic changes in the *acdh-1* knockdown group. However, the reported phenotypes were studied in *acdh-1* mutant strains (so that the developmental phase could be affected by the *acdh-1* knockout) or in concert with other mutations. Therefore, the data are not entirely comparable with our study. In humans, ACADS, which is mainly expressed in fat tissue, liver and intestine, was found to be associated with hepatocellular carcinomas (Chen et al. 2019). Furthermore, Schmidt et al. (2010) showed that misfolding of ACADS leads to increased ROS production in mitochondria and is associated with the inherited short-chain acyl-CoA dehydrogenase deficiency disorder. Due to the close, but controversial link of oxidative stress and ageing (Gems and Doonan 2009; Liguori et al. 2018; Liochev 2013), this could be a hint for a role of ACADS in the ageing process.

Thus, the measured health phenotypes in this study seem to be independently modulated, since no uniform pattern of changes after RNAi treatments was visible. Furthermore, it was shown that lifespan and healthspan are only partly correlated. The mean lifespan of nematodes with *wwp-1*, *yap-1*, *acdh-7*, and *acdh-8* knockdown correlated quite well with their pathogen resistance, whereas the increased survival during pathogenic stress *via* RNAi targeting of *frm-8, acdh-3, elo-3, ivd-1, nex-1, nex-2,* and *paxt-1* was not mirrored in their lifespan during unstressed conditions. Lifespan and stress resistance were often described as closely related, however heat and oxidative stress, but not pathogenic stress, were mainly used for these observations (Lithgow and Walker 2002; Zhou et al. 2011). Moreover, Benedetto et al. (2019) found that heat stress resistance, but not oxidative stress resistance tends to correlate with lifespan in *C. elegans*. This and an earlier study (Saul et al. 2021) show that a multiparametric approach is important in the search for genes or compounds that robustly increase healthspan as not every tested healthspan parameter may be pushed in the same direction by a given treatment. Notwithstanding differences in healthspan parameters between *C. elegans* and humans, the former may be useful to sort quickly through a number of GWAS candidate genes, provided clear homologs are available.

### Limitations of the study

The selection of candidate genes was realized according to the closest distance to the respective SNP loci. This approach is debatable, since SNPs can also affect distant genes (Claussnitzer et al. 2016). Brodie et al. (2016) showed “that affected genes are often up to 2 Mbps away from the associated SNP, and are not necessarily the closest genes to the SNP”. Thus, selecting genes which are close to the SNPs may not fully cover all potentially relevant loci. Nevertheless, Brodie and colleagues also found that “SNPs are more likely to be relevant to closer genes, but that even very distant genes are affected by the variation”. Since their described pathway-driven method to find the most relevant genes is not applicable in our study (because we aimed to find rather unknown pathways/genes associated with healthspan), the selection of the closest genes might have been the best approach for this study.

A further limitation of this study is that genes of interest were only analysed by RNAi mediated knockdown. By doing this, only SNPs that function by reducing the activity of the gene of interest were covered. But SNPs which increase the activity of the respective gene(s) should rather be analysed *via* overexpression analyses in *C. elegans,* for instance by using knock-in transgenic strains. Indeed, Baskoylu et al. (2018) showed that knock-in and knockout does not necessarily result in opposite effects. They found that SOD-1 loss and gain of function in *C. elegans* differentially contributed to the pathogenesis of Amyotrophic Lateral Sclerosis (ALS) in different neuronal populations. Thus, the involvement of a gene in health maintenance might be overlooked by analysing only the knockdown effects. In addition, it must be kept in mind that a gene whose knockdown leads to a decline of a certain healthspan parameter could have the same importance to healthspan compared to a gene with improved health after knockdown. Nevertheless, knockdown *via* RNAi was probably the most straightforward strategy to cover a decent number of genes in this first screening.

Despite the described limitations, the GWAS guided approach to identify healthspan related genes in *C. elegans* and humans is regarded as successful. This becomes evident by comparing the number of positive hits in our approach compared to genome-wide RNAi screenings. In total, seven *C. elegans* genes (*yap-1, wwp-1, paxt-1, acdh-3, acdh-7, acdh-8,* and *acdh-10*) were found to be potentially involved in health maintenance during ageing by screening 13 GWAS-based selected genes in total, which represents a hit rate of 54%. Choe and Strange (2008), for instance, screened all (about 19,000) *C. elegans* genes *via* RNAi and identified 40 genes that are essential for survival during hypertonic stress, which represents a hit rate of 0.2%. The GWAS clearly helped to narrow down the long list of *C. elegans* genes and provided potential new genes that deserve further attention, not only in *C. elegans*, but also other models.

## Conclusion

The homologs of five selected human genes, which are potentially responsible for increased healthspan as shown by a GWAS, were studied in healthspan assays in *C. elegans*. Most of the tested *C. elegans* genes seem to be involved in immune defence, but none of the homologs were shown to be overall healthspan modulators. *yap-1* and *wwp-1* (both homologs of human *WWC2*), *paxt-1* (homolog of *CDKN2AIP*) and several *acdh*-genes (homologs of *ACADS*) turned out to be particularly interesting, since their targeting via RNAi resulted in unique phenotypes regarding muscle integrity, pathogen resistance and chemotactic behaviour. The downregulation of *wwp-1* led to reduced pathogenic stress resistance and a decline in chemotaxis, but muscle quality was not impaired. RNAi targeting of *yap-1* led to improvements in sensory perception but muscle quality and pathogen resistance were not changed or even worsened. In contrast, the downregulation of *paxt-1* led to improved muscle integrity and increased pathogen resistance, but unchanged chemotactic behaviour. The knockdown of the *acdh*-genes resulted in a very heterogeneous response. RNAi targeting of *acdh-7* and *acdh-10* was detrimental for muscle integrity, but the knockdown of *acdh-7* led to an improved pathogen resistance. Knockdown of *acdh-3* improved muscle quality and pathogen resistance, whereas RNAi targeting of *acdh-8* was solely beneficial for pathogenic resistance. Further studies with additional healthspan assays and also a combined downregulation of these genes might uncover their full involvement in healthspan maintenance. In sum, the *C. elegans* assays together with the GWAS data suggest that the human genes *WWC2*, *CDKN2AIP* and *ACADS* play roles in health maintenance in older individuals.

## Supplementary Information

Below is the link to the electronic supplementary material.

Supplementary file1 (PDF 152 kb)

Supplementary file2 (XLSX 12 kb)

Supplementary file3 (PDF 195 kb)

Supplementary file4 (PDF 164 kb)

Supplementary file5 (PDF 246 kb)

Supplementary file6 (PDF 197 kb)

Supplementary file7 (PDF 165 kb)

Supplementary file8 (PDF 813 kb)

Supplementary file9 (PDF 171 kb)

Supplementary file10 (PDF 255 kb)

Supplementary file11 (ZIP 217905 kb)

## Data Availability

All data generated or analysed during this study are included in this published article and its supplementary information files.
