# Supplementary material for: Identification of healthspan-promoting genes in Caenorhabditis elegans based on a human GWAS study
Source: Biogerontology. 2022 Jun 24;23(4):431–52. doi: 10.1007/s10522-022-09969-8 (PMC9388463; doi:10.1007/s10522-022-09969-8)
Supplement: Supplementary file 1 — Supplementary file1 (PDF 152 kb) [file 10522_2022_9969_MOESM1_ESM.pdf]

Title: Identification of healthspan-promoting genes in *Caenorhabditis elegans* based on a human GWAS study

Journal: Biogerontology

Authors: Nadine Saul, Ineke Dhondt, Mikko Kuokkanen, Markus Perola, Clara Verschuuren, Brecht Wouters, Henrik von Chrzanowski, Winnok H. De Vos, Liesbet Temmerman, Walter Luyten, Aleksandra Zečić, Tim Loier, Christian Schmitz-Linneweber, Bart P. Braeckman

Corresponding author: Nadine Saul, Molecular Genetics Group, Institute of Biology, Humboldt University of Berlin, 10115 Berlin, Germany; Email: nadine.saul@gmx.de

ESM_1: Scanning of medical reimbursement records

Death: all-cause mortality.
Death age: all-cause mortality age.

HILMO, The Hospital Discharge Register.
S_PAADG, Main diagnosis (symptom)
S_DG2, 1st side diag (symptom)
S_DG3, 2nd side diag (symptom)
S_DG4, 3rd side diag (ICD-8/9 only)
S_PAADGE, Main diagnosis (cause)
S_DG2E, 1st side diag (cause)
S_DG3E, 2nd side diag (cause)

Kela, Reimbursements for medicine expenses.
Causes of death:
k_tpks, Underlying cause of death
k_vks, Immediate cause of death
k_m1, 1st contributing c.o.d.
k_m2, 2nd contributing c.o.d.
k_m3, 3rd contributing c.o.d.

DISEASES, prevalent or incident.

Display name: Cancers.
Keywords: abnormal cells divide and invade nearby tissues, neoplasms. **Protocol:** HILMO: C00-C43, C45-C97 (ICD-10) 140-172, 174-208 (ICD-8/9) as S_PAADG, S_PAADGE, S_DG2, S_DG2E, S_DG3, S_DG3E, S_DG4. CAUSES OF DEATH: Like Hilmo: as k_tpks, k_vks, k_m1, k_m2, k_m3.

Display name: Diabetes.
Keywords: endocrine, nutritional and metabolic diseases, glucose intolerance, insulin, E10 (ICD_10), E11 (ICD-10), E12 (ICD-10), E13 (ICD-13), E14 (ICD-10), 250 (ICD-9), 250 (ICD-9), KELA code 103, ATC class A10.

Display name: Dementia.
Keywords: brain disorders, loss of intellectual abilities, Alzheimer's disease, mental disorder, F00 (ICD-10), F01 (ICD-10), F02 (ICD-10), F03 (ICD-10), G30 (ICD-10), 3310 (ICD-9), 4378A (ICD-9), 290 (ICD-9), 290 (ICD-8), KELA code 307, ATC class N06D.

Display name: Chronic obstructive pulmonary disease. **Keywords:** respiratory system, COPD, J43 (ICD-10), J44 (ICD-10), 491 (ICD-9), 492 (ICD-9), 491 (ICD-8), 492 (ICD-8).

Display name: Asthma. **Keywords:** autoimmune, chronic obstructive lung disease, bronchial disorder, chronic lower respiratory diseases, J45 (ICD-10), J46 (ICD-10), 493 (ICD-8), 493 (ICD-9), KELA code 203.

Display name: Crohn's disease. **Keywords:** autoimmune, inflammation of the digestive system, noninfective enteritis and colitis, K50 (ICD-10), 555 (ICD-9), 5630 (ICD-8), KELA code 208. **Protocol:** HILMO: K50 (ICD-10); 555 (ICD-8/9) as S_PAADG, S_PAADGE, S_DG2, S_DG2E, S_DG3, S_DG3E, S_DG4. CAUSES OF DEATH: Like Hilmo: as k_tpks, k_vks, k_m1, k_m2, k_m3. KELA reimbursed medication: IBD (KELA code 208).

Display name: Malabsorption. **Keywords:** autoimmune, decreased absorption of nutrients in the gastrointestinal tract, K90 (ICD-10), K91 (ICD-10), 579 (ICD-8), 579 (ICD-9). **Protocol:** HILMO: K90, K91 (ICD-10); 579 (ICD-8/9) as S_PAADG, S_PAADGE, S_DG2, S_DG2E, S_DG3, S_DG3E, S_DG4. CAUSES OF DEATH: Like Hilmo: as k_tpks, k_vks, k_m1, k_m2, k_m3.

Display name: Rheuma. **Keywords:** autoimmune, inflammatory polyarthropathies, joints, metabolic derangement, disorder of connective tissue, M05 (ICD-10), M06 (ICD-10), M07 (ICD-10), M08 (ICD-10), M09 (ICD-10), M10 (ICD-10), M11 (ICD-10), M12 (ICD-10), M13 (ICD-10), M32 (ICD-10), M33 (ICD-10), M45 (ICD-10), 710 (ICD-9), 714 (ICD-9), 420 (ICD-9), 725 (ICD-9), 712 (ICD-8), 734 (ICD-8), KELA code 202.

Display name: Chronic kidney failure. **Keywords:** genitourinary system, renal failure, N18 (ICD-10), N19 (ICD-10), 585 (ICD-9), 58200 (ICD-8). **Protocol:** HILMO: N18–N19 (ICD-10); 585 (ICD-9); 58200 (ICD-8) as S_PAADG, S_PAADGE, S_DG2, S_DG2E, S_DG3, S_DG3E, S_DG4. CAUSES OF DEATH: Like Hilmo: as k_tpks, k_vks, k_m1, k_m2, k_m3.

Display name: Myocardial infarction. **Keywords:** cvd, myocardial necrosis, blood supply interruption, circulatory system, I21 (ICD-10), I22 (ICD-10), 410 (ICD-9), 410 (ICD-8). **Protocol:** HILMO: I21, I22 (ICD-10); 410 (ICD-8/9) as S_PAADG, S_PAADGE, S_DG2, S_DG2E, S_DG3, S_DG3E, S_DG4. CAUSES OF DEATH I21, I22 (ICD-10); 410 (ICD-8/9) as k_tpks, k_vks, k_m1, k_m2, k_m3.

Display name: Major coronary heart disease event. **Keywords:** cvd, narrowing or blockage of the coronary arteries, ischaemic heart disease, circulatory system, I200 (ICD-10), I21 (ICD-10), I22 (ICD-10), 410 (ICD-9), 4110 (ICD-9), 410 (ICD-8), 4110 (ICD-8).

Display name: Ischaemic heart disease. **Keywords:** cvd, myocardial ischemia, ischaemic heart disease, circulatory system, I20 (ICD-10), I21 (ICD-10), I22 (ICD-10), I23 (ICD-10), I24 (ICD-10), I25 (ICD-10), 410 (ICD-9), 411 (ICD-9), 412 (ICD-9), 413 (ICD-9), 414 (ICD-9), 410 (ICD-8), 411 (ICD-8), 412 (ICD-8), 413 (ICD-8), 414 (ICD-8), KELA code 206.

Display name: Cardiovascular diseases. **Keywords:** cvd, circulatory system. **Protocol:** Major coronary heart disease event (CHD), or Stroke, excluding SAH (STR) - whichever of these happens first.

Display name: Stroke, ex SAH. **Comments:** Note: includes intracerebral haemorrhage. **Keywords:** 434 (ICD-8), 436 (ICD-8), cvd, blood flow to brain stops, cerebrovascular disorder, circulatory system, I61 (ICD-10), I63 (ICD-10), 431 (ICD-9), 4330A (ICD-9), 4331A (ICD-9), 4339A (ICD-9), 4340A (ICD-9), 4341A (ICD-9), 4349A (ICD-9), 436 (ICD-9), 431 (ICD-8) (except 43101, 43191), 433 (ICD-8).

Display name: Stroke, inc SAH. **Comments:** Note: also includes intracerebral haemorrhage. **Keywords:** cvd, blood flow to brain stops, cerebrovascular disorder, circulatory system, I60 (ICD-10), I61 (ICD-10), I63 (ICD-10), I64 (ICD-10), 430 (ICD-9), 431 (ICD-9), 4330A (ICD-9), 4331A (ICD-9), 4339A (ICD-9), 4340A (ICD-9), 4341A (ICD-9), 4349A (ICD-9), 436 (ICD-9), 430 (ICD-8), 431 (ICD-8) (except 43101, 43191), 433 (ICD-8), 434 (ICD-8), 436 (ICD-8). **Protocol:** HILMO: I60-I64 (not I636) (ICD-10); 430, 431, 4330A, 4331A, 4339A, 4340A, 4341A, 4349A, 436 (ICD-9); 430, 431 (except 43101, 43191) 433, 434, 436 (ICD-8). CAUSES OF DEATH: Like Hilmo: as k_tpks, k_vks, k_m1, k_m2, k_m3.

Display name: Ischaemic stroke, ex all haemorrhages. **Keywords:** cvd, cerebrovascular disorder, circulatory system, I63 (ICD-10), I64 (ICD-10), 4330A (ICD-9), 4331A (ICD-9), 4339A (ICD-9), 4340A (ICD-9), 4341A (ICD-9), 4349A (ICD-9), 436 (ICD-9), 433 (ICD-8), 434 (ICD-8), 436 (ICD-8). **Protocol:** HILMO: I63-I64 (not I636) (ICD-10); 4330A, 4331A, 4339A, 4340A, 4341A, 4349A, 436 (ICD-9); 433, 434, 436 (ICD-8) as S_PAADG, S_PAADGE, S_DG2, S_DG2E, S_DG3, S_DG3E, S_DG4. CAUSES OF DEATH: Like Hilmo: as k_tpks, k_vks, k_m1, k_m2, k_m3.

Display name: Heart failure. **Keywords:** cvd, inability of the heart to pump blood at an adequate rate, circulatory system, I50 (ICD-10), I110 (ICD-10), I130 (ICD-10), I132 (ICD-10), 4029B (ICD-9), 4148 (ICD-9), 428 (ICD-9), 42700 (ICD-8), 42710 (ICD-8), 428 (ICD-8), KELA code 201, ATC code C03CA01, ATC code C03EB01.

Display name: Major adverse cardiovascular event. **Keywords:** cvd, circulatory system. **Protocol:** Cardiovascular disease (CVD), or Heart failure (HFAIL) - whichever of these happens first
