# Supplementary material for: Identification of healthspan-promoting genes in Caenorhabditis elegans based on a human GWAS study
Source: Biogerontology. 2022 Jun 24;23(4):431–52. doi: 10.1007/s10522-022-09969-8 (PMC9388463; doi:10.1007/s10522-022-09969-8)
Supplement: Supplementary file 3 — Supplementary file3 (PDF 195 kb) [file 10522_2022_9969_MOESM3_ESM.pdf]

Title: Identification of healthspan-promoting genes in *Caenorhabditis elegans* based on a human GWAS study

Journal: Biogerontology

Authors: Nadine Saul, Ineke Dhondt, Mikko Kuokkanen, Markus Perola, Clara Verschuuren, Brecht Wouters, Henrik von Chrzanowski, Winnok H. De Vos, Liesbet Temmerman, Walter Luyten, Aleksandra Zečić, Tim Loier, Christian Schmitz-Linneweber, Bart P. Braeckman

Corresponding author: Nadine Saul, Molecular Genetics Group, Institute of Biology, Humboldt University of Berlin, 10115 Berlin, Germany; Email: nadine.saul@gmx.de

ESM_3: Overview of *C. elegans* strains used in this study

Strain	Genotype	Reports for	Positive controls to validate reporter strain assays
**N2 Bristol**	*wild type*	-	-
**RW1596**	*myo-3(st386); stEx30 [myo-3p::GFP::myo-3 + rol-6(su1006)])*		
**GA410**	*wuls56[sod-3p::GFP::pRF4(rol-6(su1006))]*	DAF-16	Heat stress (2h at 37°C)
**TJ375**	*gpIs1[hsp-16-2p::GFP]*	HSF-1	Heat stress (2h at 37°C)
**SJ4100**	*zcIs13[hsp-6p::GFP]*	mtUPR	*nuo-2* RNAi
**SJ4005**	*zcIs4 [hsp-4p::GFP]*	erUPR	*mdt-15* RNAi
