# Supplementary material for: Identification of healthspan-promoting genes in Caenorhabditis elegans based on a human GWAS study
Source: Biogerontology. 2022 Jun 24;23(4):431–52. doi: 10.1007/s10522-022-09969-8 (PMC9388463; doi:10.1007/s10522-022-09969-8)
Supplement: Supplementary file 4 — Supplementary file4 (PDF 164 kb) [file 10522_2022_9969_MOESM4_ESM.pdf]

Title: Identification of healthspan-promoting genes in *Caenorhabditis elegans* based on a human GWAS study

Journal: Biogerontology

Authors: Nadine Saul, Ineke Dhondt, Mikko Kuokkanen, Markus Perola, Clara Verschuuren, Brecht Wouters, Henrik von Chrzanowski, Winnok H. De Vos, Liesbet Temmerman, Walter Luyten, Aleksandra Zečić, Tim Loier, Christian Schmitz-Linneweber, Bart P. Braeckman

Corresponding author: Nadine Saul, Molecular Genetics Group, Institute of Biology, Humboldt University of Berlin, 10115 Berlin, Germany; Email: nadine.saul@gmx.de

ESM_4: Details for SNPs with highest significance

CHR	SNP	Position (hg19)	Other allele	Effect allele	Info	Cases	Controls	N	EAF	SE	Beta	P	Closest genes	GWAS
9	rs11143626	76202111	C	G	0.93	750	2663	3413	0.48	0.07	0.33	2.9e-07	ANXA1	2
12	rs7964228	121179277	G	A	0.99	750	2663	3413	0.03	0.24	-1.08	2.9e-07	ACADS	2
4	rs56392732	184336096	C	T	0.91	750	2663	3413	0.06	0.17	-0.79	6.5e-07	WWC2-CLDN22/24-CDKN2AIP	2
21	rs1123415	43875340	G	A	0.97	750	2663	3413	0.32	0.07	0.33	1.4e-06	UBASH3A-RSPH1	2
4	rs74705824	111155582	C	G	0.97	750	2663	3413	0.05	0.13	0.65	2.3e-06	ELOVL6	2
1	rs3205087	3800242	A	G	0.98	750	2663	3413	0.36	0.06	0.31	2.4e-06	DFFB	2
21	rs1123415	43875340	G	A	0.97	750	1502	2252	0.32	0.07	0.35	1.1e-06	UBASH3A-RSPH1	1
4	rs74705824	111155582	C	G	0.97	750	1502	2252	0.05	0.14	0.69	2.5e-06	ELOVL6	1
12	rs7964228	121179277	G	A	0.99	750	1502	2252	0.03	0.24	-1.02	3.5e-06	ACADS	1
9	rs11143626	76202111	C	G	0.93	750	1502	2252	0.49	0.07	0.30	5.8e-06	closest ANXA1	1
1	rs3205087	3800242	A	G	0.98	750	1502	2252	0.37	0.07	0.31	6.0e-06	DFFB	1
4	rs56392732	184336096	C	T	0.91	750	1502	2252	0.05	0.17	-0.64	7.9e-05	WWC2-CLDN22/24-CDKN2AIP	1
