# Supplementary material for: Identification of healthspan-promoting genes in Caenorhabditis elegans based on a human GWAS study
Source: Biogerontology. 2022 Jun 24;23(4):431–52. doi: 10.1007/s10522-022-09969-8 (PMC9388463; doi:10.1007/s10522-022-09969-8)
Supplement: Supplementary file 5 — Supplementary file5 (PDF 246 kb) [file 10522_2022_9969_MOESM5_ESM.pdf]

Title: Identification of healthspan-promoting genes in *Caenorhabditis elegans* based on a human GWAS study

Journal: Biogerontology

Authors: Nadine Saul, Ineke Dhondt, Mikko Kuokkanen, Markus Perola, Clara Verschuuren, Brecht Wouters, Henrik von Chrzanowski, Winnok H. De Vos, Liesbet Temmerman, Walter Luyten, Aleksandra Zečić, Tim Loier, Christian Schmitz-Linneweber, Bart P. Braeckman

Corresponding author: Nadine Saul, Molecular Genetics Group, Institute of Biology, Humboldt University of Berlin, 10115 Berlin, Germany; Email: nadine.saul@gmx.de

ESM_5: Selection of 13 *C. elegans* genes potentially involved in healthy ageing

Human gene	*C. elegans* homologs	NCBI HomoloGene	Ortholist 2	HMMER	Aceview
DFFB	/	/	/	/	/
ELOVL6	***elo-3***	✓	✓	✓	✓
	elo-9	/	✓	✓	/
	elo-4	/	✓	✓	/
	elo-1	/	✓	✓	/
	elo-2	/	✓	✓	/
	elo-5	/	/	✓	/
	elo-6	/	/	✓	/
	elo-7	/	/	✓	/
WWC2	***wwp-1***	/	/	✓	/
	Y92H12A.2	/	/	✓	/
	magi-1	/	/	✓	/
	frm-8	/	/	✓	/
	yap-1	/	/	✓	/
	sav-1	/	/	✓	/
	gex-2	/	/	/	✓
CDKN2AIP	***paxt-1***	/	/	✓	✓
ANXA1	***nex-1***	/	✓	✓	/
	nex-2	/	✓	✓	✓
	nex-3	/	/	✓	/
	nex-4	/	/	✓	/
ACADS	***acdh-3***	/	✓	✓	✓
	acdh-1	/	✓	✓	✓
	ivd-1	/	/	✓	✓
	acdh-10	/	/	✓	✓
	acdh-8	/	/	✓	✓
	acdh-7	/	/	✓	✓
	acdh-4	/	/	✓	/
	acdh-9	/	/	✓	/
	acdh-2	/	/	✓	/
	acdh-5	/	/	✓	/

	acdh-6	/	/	✓	/
	acdh-12	/	/	✓	/
	F54D5.7	/	/	✓	/
	acdh-11	/	/	✓	/
	acdh-13	/	/	✓	/
CLDN22	/	/	/	/	/
CLDN24	/	/	/	/	/
	T07F12.1	/	✓	✓	✓
	C52E4.7	/	✓	✓	/
	F53B6.7	/	✓	✓	/
UBASH3A	*F55A11.11*	/	✓	✓	/
	F09C12.8	/	✓	✓	/
	ZK484.6	/	/	✓	/
	sorb-1	/	/	✓	/
RSPH1	*jph-1*	/	/	✓	✓

Abbreviations: DFFB = DNA Fragmentation Factor Subunit Beta, ELOVL6 = Elongation of very long chain fatty acids protein 6, WWC2 = WW and C2 domain containing 2, CDKN2AIP = CDKN2A Interacting Protein, ANXA1 = Annexin A1, ACADS = Acyl-CoA dehydrogenase, C-2 to C-3 short chain, CLDN22/24 = claudin 22/24, UBASH3A = ubiquitin associated and SH3 domain containing A, RSPH1 = radial spoke head component 1.
