# Supplementary material for: Identification of healthspan-promoting genes in Caenorhabditis elegans based on a human GWAS study
Source: Biogerontology. 2022 Jun 24;23(4):431–52. doi: 10.1007/s10522-022-09969-8 (PMC9388463; doi:10.1007/s10522-022-09969-8)
Supplement: Supplementary file 6 — Supplementary file6 (PDF 197 kb) [file 10522_2022_9969_MOESM6_ESM.pdf]

Title: Identification of healthspan-promoting genes in *Caenorhabditis elegans* based on a human GWAS study

Journal: Biogerontology

Authors: Nadine Saul, Ineke Dhondt, Mikko Kuokkanen, Markus Perola, Clara Verschuuren, Brecht Wouters, Henrik von Chrzanowski, Winnok H. De Vos, Liesbet Temmerman, Walter Luyten, Aleksandra Zečić, Tim Loier, Christian Schmitz-Linneweber, Bart P. Braeckman
Corresponding author: Nadine Saul, Molecular Genetics Group, Institute of Biology, Humboldt University of Berlin, 10115 Berlin, Germany; Email: nadine.saul@gmx.de

ESM_6: Pathogen-stress survival characteristics during RNAi treatment

treatment	n	mean survival (days)	SEM	days until deaths of population reached				
				25 %	50 %	75 %	90 %	100 %
**Pathogen exposure initiation: 3rd day of adulthood**								
**EV**	259	5.77	0.1	4.51	5.39	6.36	7.09	9
acdh-1	107	5.07	0.21	4.06*	4.94*	5.91	6.89	9
acdh-3	108	5.42	0.25	3.4	5.35	6.9*	8.04*	10
acdh-7	108	5.77	0.17	4.42	5.25	6.41	7.38	10
acdh-8	104	6.4*	0.17	5.13*	6.18*	6.94*	7.77	9
acdh-10	106	5.44	0.21	4.22	5.03	6.28	7.5	10
elo-3	110	6.2	0.13	4.85	5.66	6.56	7.42	9
frm-8	99	6.36*	0.14	5.13*	6*	6.85*	7.54	9
ivd-1	106	5.16	0.2	4.08*	4.97*	5.94	6.9	9
nex-1	109	6.47*	0.15	5.2*	5.85	6.84	8.11*	10
nex-2	91	5.71	0.2	4.42	5.48	6.51	7.32	9
paxt-1	107	5.48	0.18	4.45	5.39	6.13	6.78	9
wwp-1	112	4.36*	0.14	3.34*	4.22*	4.72*	5.18*	7
yap-1	98	4.64*	0.16	3.2*	4.27*	5.16	5.96	8
**Pathogen exposure initiation: 7th day of adulthood**								
**EV**	230	4.63	0.13	2.67	4.44	5.44	6.45	10
acdh-1	100	4.72	0.22	2.2	4.71*	6*	6.88	9
acdh-3	101	5.6*	0.2	4.4*	5.56*	6.42*	6.96	10
acdh-7	105	5.32*	0.2	3.85	5.2*	6.31*	6.94	9
acdh-8	111	5.59*	0.15	4.35*	5.38*	6.08*	6.86	9
acdh-10	105	4.78	0.22	1.93	4.61	5.9	7.08	10
elo-3	105	5.54*	0.21	4.08	5.41*	6.46*	7.5*	10
frm-8	108	5.26*	0.19	3.33	5.21*	6.19*	6.96	10
ivd-1	106	5.31*	0.2	3.5	5.28*	6.25*	6.97	10
nex-1	106	5.78*	0.19	4.45*	5.54*	6.48*	7.26*	12
nex-2	101	5.46*	0.22	3.79	5.4*	6.4*	6.97	10
paxt-1	106	5.59*	0.2	4.13*	5.38*	6.42*	7.44*	10
wwp-1	59	3.41*	0.12	2.28*	2.9*	3.58*	4.02	5
yap-1	96	4.43	0.17	2.64	4.29	5.14*	5.73	10

Differences compared to control were considered significant at $p < 0.05$ (*). p-value determination was realized with log-rank test and subsequent Bonferroni correction for the mean lifespan and Fisher's Exact Test for specific time points. No significances were analysed for the time point of 100% deaths.
