# Supplementary material for: Identification of healthspan-promoting genes in Caenorhabditis elegans based on a human GWAS study
Source: Biogerontology. 2022 Jun 24;23(4):431–52. doi: 10.1007/s10522-022-09969-8 (PMC9388463; doi:10.1007/s10522-022-09969-8)
Supplement: Supplementary file 7 — Supplementary file7 (PDF 165 kb) [file 10522_2022_9969_MOESM7_ESM.pdf]

Title: Identification of healthspan-promoting genes in *Caenorhabditis elegans* based on a human GWAS study

Journal: Biogerontology
Authors: Nadine Saul, Ineke Dhondt, Mikko Kuokkanen, Markus Perola, Clara Verschuuren, Brecht Wouters, Henrik von Chrzanowski, Winnok H. De Vos, Liesbet Temmerman, Walter Luyten, Aleksandra Zečić, Tim Loier, Christian Schmitz-Linneweber, Bart P. Braeckman
Corresponding author: Nadine Saul, Molecular Genetics Group, Institute of Biology, Humboldt University of Berlin, 10115 Berlin, Germany; Email: nadine.saul@gmx.de

ESM_7: Pathogen-stress survival characteristics during RNAi treatment (internal controls)

treatment	n	mean survival (days)	SEM	days until deaths of population reached				
				25 %	50 %	75 %	90 %	100 %
**Pathogen exposure initiation: 3rd day of adulthood**								
**EV**	186	5.67	0.07	4.56	5.27	5.8	6.43	8
daf-2	86	10.92*	0.19	9.48*	10.38*	11.54*	12.62*	14
daf-16	93	5.1*	0.09	3.97*	4.59*	5.29*	5.8	7
**Pathogen exposure initiation: 7th day of adulthood**								
**EV**	183	5.88	0.1	4.65	5.55	6.37	6.87	9
daf-2	104	11.04*	0.25	9.25*	10.94*	12.43*	13.45*	15
daf-16	95	3.09*	0.09	2.02*	2.52*	3.07*	3.86*	5

Differences compared to control were considered significant at $p < 0.05$ (*). p-value determination was realized with log-rank test and subsequent Bonferroni correction for the mean lifespan and Fisher's Exact Test for specific time points. No significances were analysed for the time point of 100% deaths.
