# Supplementary material for: Identification of healthspan-promoting genes in Caenorhabditis elegans based on a human GWAS study
Source: Biogerontology. 2022 Jun 24;23(4):431–52. doi: 10.1007/s10522-022-09969-8 (PMC9388463; doi:10.1007/s10522-022-09969-8)
Supplement: Supplementary file 8 — Supplementary file8 (PDF 813 kb) [file 10522_2022_9969_MOESM8_ESM.pdf]

Title: Identification of healthspan-promoting genes in *Caenorhabditis elegans* based on a human GWAS study

Journal: Biogerontology

Authors: Nadine Saul, Ineke Dhondt, Mikko Kuokkanen, Markus Perola, Clara Verschuuren, Brecht Wouters, Henrik von Chrzanowski, Winnok H. De Vos, Liesbet Temmerman, Walter Luyten, Aleksandra Zečić, Tim Loier, Christian Schmitz-Linneweber, Bart P. Braeckman

Corresponding author: Nadine Saul, Molecular Genetics Group, Institute of Biology, Humboldt University of Berlin, 10115 Berlin, Germany; Email: nadine.saul@gmx.de

ESM_8: Supplementary figures S1, S2 & S3 (survival curves)

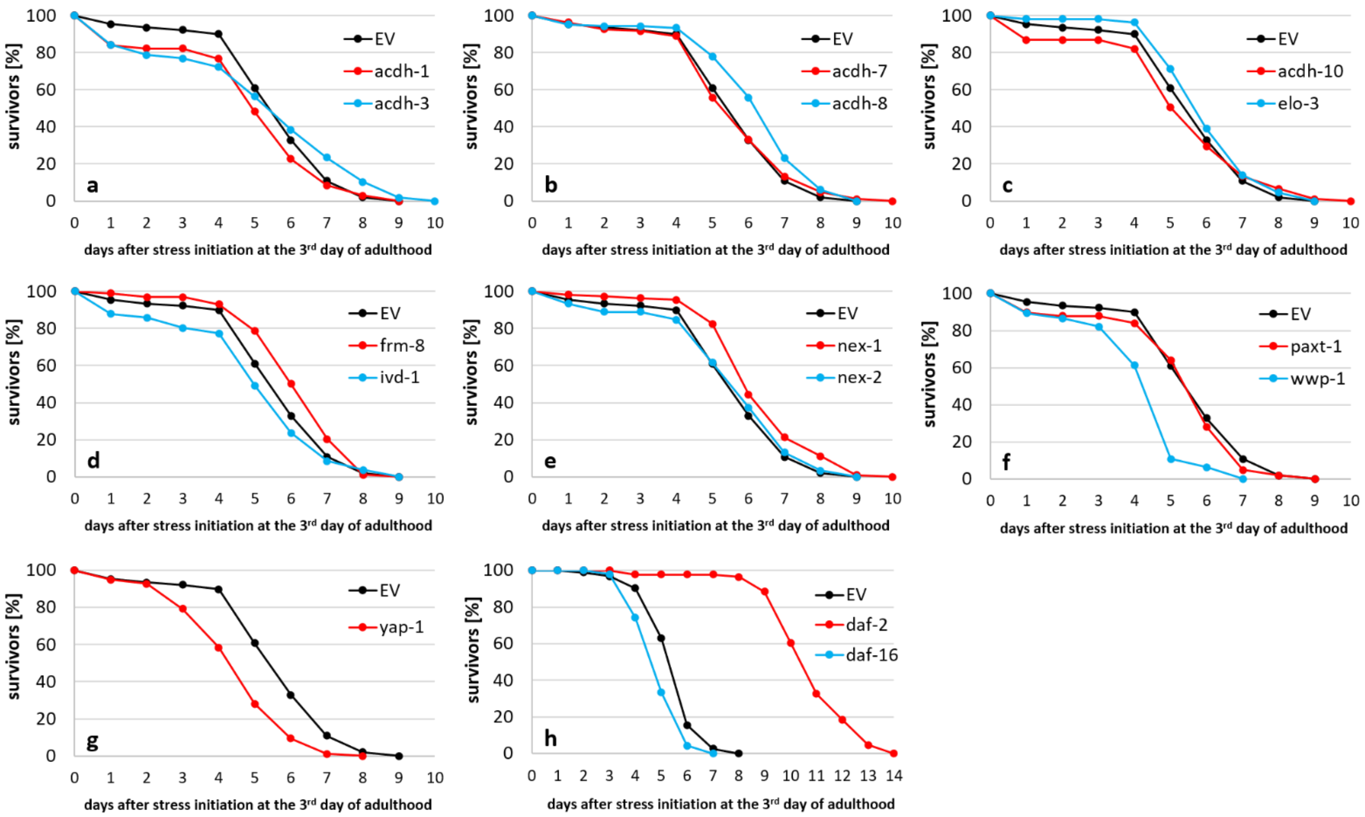

Figure S1: Survival during pathogenic stress starting at the 3rd day of adulthood
Nematodes at the L4 stage were treated with 13 different RNAi strains. The empty vector (EV) strain was used as control. Starting at the 3rd day of adulthood, they were exposed to the pathogen *P. luminescens* and their survival was monitored. Survival curves are shown for nematodes treated with RNAi targeting *acdh-1* & *acdh-3* (**a**), *acdh-7* & *acdh-8* (**b**), *acdh-10* & *elo-3* (**c**), *frm-8* & *ivd-1* (**d**), *nex-1* & *nex-2* (**e**), *paxt-1* & *wwp-1* (**f**), *yap-1* (**g**), and *daf-2* & *daf-16* (**h**).

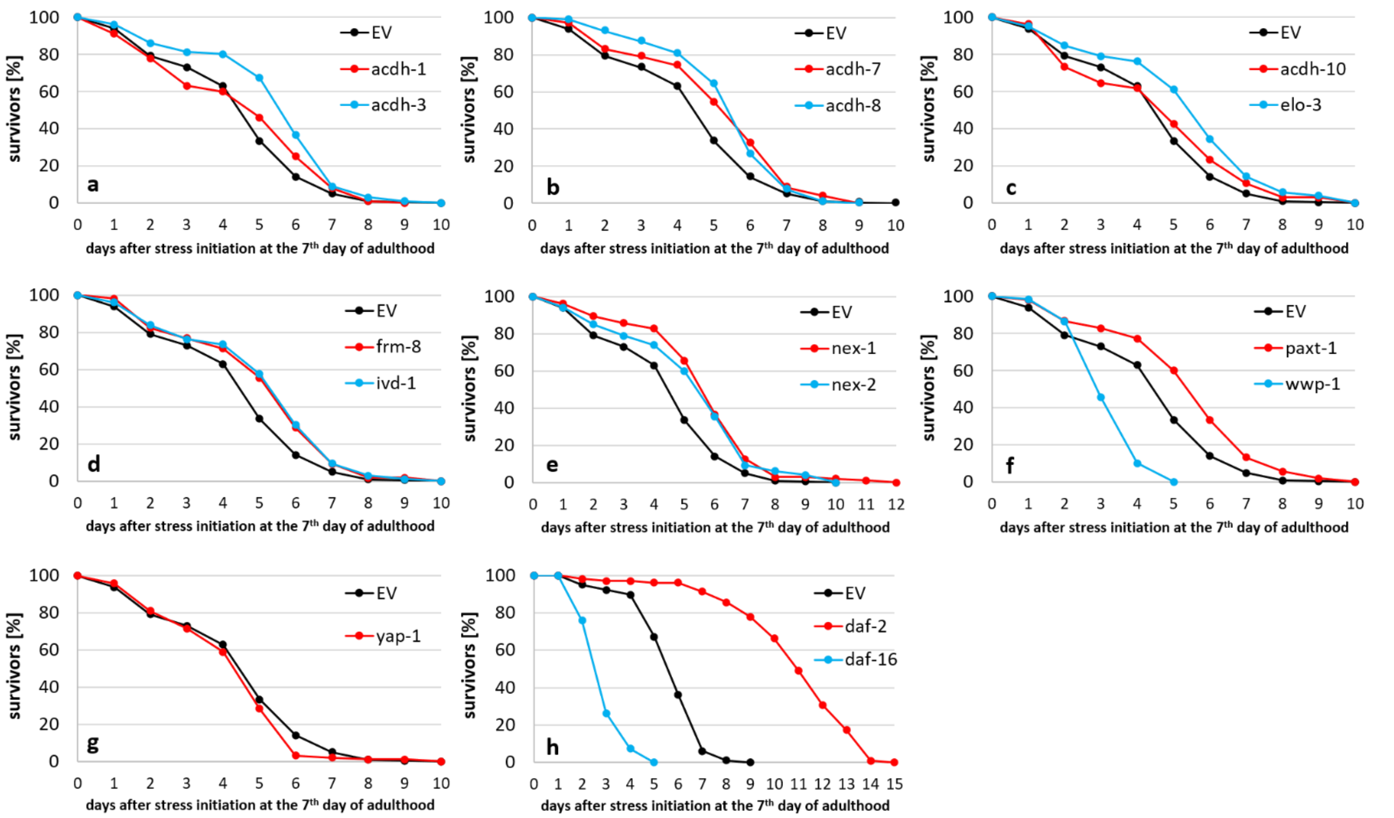

Figure S2: Survival during pathogenic stress starting at the 7th day of adulthood

Nematodes at the L4 stage were treated with 13 different RNAi strains. The empty vector (EV) strain was used as control. Starting at the 7th day of adulthood, they were exposed to the pathogen *P. luminescens* and their survival was monitored. Survival curves are shown for nematodes treated with RNAi targeting *acdh-1* & *acdh-3* (**a**), *acdh-7* & *acdh-8* (**b**), *acdh-10* & *elo-3* (**c**), *frm-8* & *ivd-1* (**d**), *nex-1* & *nex-2* (**e**), *paxt-1* & *wwp-1* (**f**), *yap-1* (**g**), and *daf-2* & *daf-16* (**h**).

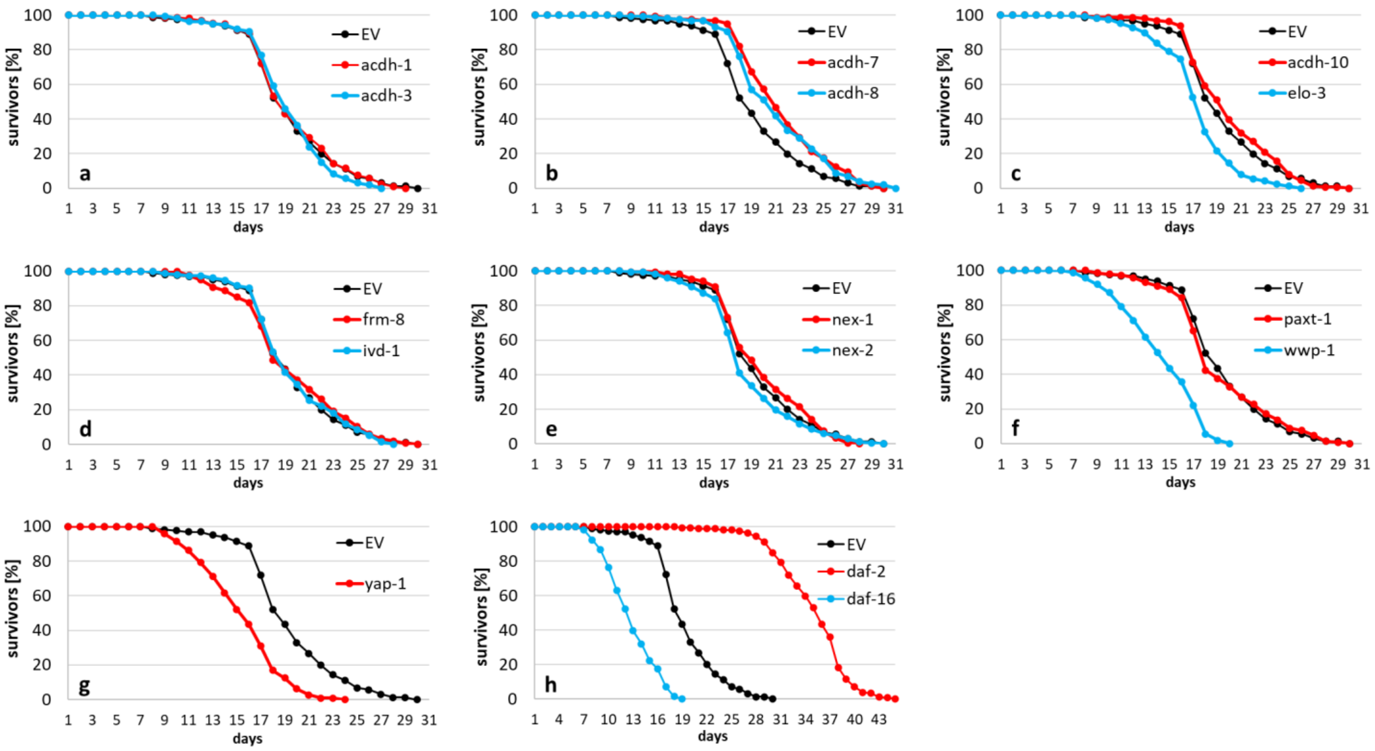

Figure S3: Lifespan of RNAi treated neamtodes

Nematodes at the L4 stage were treated with 13 different RNAi strains. The empty vector (EV) strain was used as control. Their survival was monitored starting with the 1st day of adulthood. Survival curves are shown for nematodes treated with RNAi targeting *acdh-1* & *acdh-3* (**a**), *acdh-7* & *acdh-8* (**b**), *acdh-10* & *elo-3* (**c**), *frm-8* & *ivd-1* (**d**), *nex-1* & *nex-2* (**e**), *paxt-1* & *wwp-1* (**f**), *yap-1* (**g**), and *daf-2* & *daf-16* (**h**).
