# Supplementary material for: Identification of healthspan-promoting genes in Caenorhabditis elegans based on a human GWAS study
Source: Biogerontology. 2022 Jun 24;23(4):431–52. doi: 10.1007/s10522-022-09969-8 (PMC9388463; doi:10.1007/s10522-022-09969-8)
Supplement: Supplementary file 9 — Supplementary file9 (PDF 171 kb) [file 10522_2022_9969_MOESM9_ESM.pdf]

Title: Identification of healthspan-promoting genes in *Caenorhabditis elegans* based on a human GWAS study

Journal: Biogerontology
Authors: Nadine Saul, Ineke Dhondt, Mikko Kuokkanen, Markus Perola, Clara Verschuuren, Brecht Wouters, Henrik von Chrzanowski, Winnok H. De Vos, Liesbet Temmerman, Walter Luyten, Aleksandra Zečić, Tim Loier, Christian Schmitz-Linneweber, Bart P. Braeckman
Corresponding author: Nadine Saul, Molecular Genetics Group, Institute of Biology, Humboldt University of Berlin, 10115 Berlin, Germany; Email: nadine.saul@gmx.de

ESM_9: Lifespan characteristics during RNAi treatment

treatment	n	mean lifespan (days)	SEM	days until deaths of population reached				
				25 %	50 %	75 %	90 %	100 %
**EV**	167	19.48	0.3	16.82	18.25	21.25	24.27	30
daf-2	161	35.09*	0.34	31.57*	35.3*	37.62*	39.3*	45
daf-16	158	12.88*	0.24	10.11*	12.18*	14.7*	16.73*	19
acdh-1	160	19.62	0.3	16.83	18.31	21.69	24.38	29
acdh-3	167	19.38	0.26	17.1	18.69	20.91	22.74	27
acdh-7	167	21.66*	0.29	18.48*	20.69*	23.53*	26.79	30
acdh-8	164	21.23*	0.31	18.06*	20.13*	23.64*	25.86	31
acdh-10	165	20.13	0.28	16.9	19.08	22.33	24.72	30
elo-3	166	17.52*	0.25	15.93*	17.12*	18.69*	20.67*	26
frm-8	168	19.49	0.33	16.5	17.94	22.14	25.03	30
ivd-1	164	19.58	0.3	16.84	18.28	21.1	24.52	28
nex-1	168	19.94	0.29	16.9	18.78	22.25*	24.61	28
nex-2	170	18.85	0.29	16.45	17.61	20.19	23.53	30
paxt-1	151	19.29	0.34	16.48	17.67	21.42	24.77	30
wwp-1	163	14.47*	0.25	11.52	14.3	16.78	17.73	20
yap-1	170	15.52*	0.31	12.53*	15.25	17.43	19.39*	24

Differences compared to control were considered significant at $p < 0.05$ (*). p-value determination was realized with log-rank test and subsequent Bonferroni correction for the mean lifespan and Fisher's Exact Test for specific time points. No significances were analysed for the time point of 100% deaths.
