# Supplementary material for: Identification of healthspan-promoting genes in Caenorhabditis elegans based on a human GWAS study
Source: Biogerontology. 2022 Jun 24;23(4):431–52. doi: 10.1007/s10522-022-09969-8 (PMC9388463; doi:10.1007/s10522-022-09969-8)
Supplement: Supplementary file 10 — Supplementary file10 (PDF 255 kb) [file 10522_2022_9969_MOESM10_ESM.pdf]

Title: Identification of healthspan-promoting genes in *Caenorhabditis elegans* based on a human GWAS study

Journal: Biogerontology
Authors: Nadine Saul, Ineke Dhondt, Mikko Kuokkanen, Markus Perola, Clara Verschuuren, Brecht Wouters, Henrik von Chrzanowski, Winnok H. De Vos, Liesbet Temmerman, Walter Luyten, Aleksandra Zečić, Tim Loier, Christian Schmitz-Linneweber, Bart P. Braeckman
Corresponding author: Nadine Saul, Molecular Genetics Group, Institute of Biology, Humboldt University of Berlin, 10115 Berlin, Germany; Email: nadine.saul@gmx.de

ESM_10: Verification of RNAi efficiency via RT-qPCR

To verify the sustaining effect of RNAi via feeding bacteria in *C. elegans* a RT-qPCR was performed. Nematodes at the L4 stage were treated with the respective RNAi strain or EV as a control. At the 3rd day of adulthood, four individuals were transferred into 4 µl of freshly prepared Worm Lysis buffer (5 mM Tris, pH 8.0; 0.5% Triton X-100; 0.5% Tween 20; 0.25 mM EDTA; 1 mg/ml proteinase K; dd H2O) in a PCR tube. Three samples were prepared per treatment group and nine samples for the EV-control. cDNA was synthesized according to Ly *et al.* (2015) and the manufacturer's manual for the Maxima H Minus First Strand cDNA Synthesis Kit (Thermo Scientific). A negative control (-RT) was created for each sample by substituting reverse transcriptase by water.

All primers were selected with the help of NCBI (https://www.ncbi.nlm.nih.gov/tools/primer-blast/) and primer sequences as well as the used annealing temperatures are summarized in **Table S1**. qPCR was performed with the Luna Universal qPCR Master Mix (New England BioLabs) according to manufacturer's manual as well as with the MyiQ Single Color RT-PCR Detection System from BioRad and the iQ5 Optical System Software version 2.1 (BioRad). For the relative quantification, 2 µl of each cDNA (or -RT) preparation were added to 18 µl of the master mix including 250 nM of the respective primer. Each cDNA sample was measured in technical triplicates. After initial denaturation at 95°C for 2 min, 40 cycles were performed with 95°C (10 sec), 54 °C-60 °C (20 sec) and 72°C (45 sec). All PCR products were tested via gel electrophoresis and melt curve analysis. No template and -RT-controls were used as negative controls. In addition to the genes of interest, the reference genes *act-1* and *cdc-42* were also amplified via RT-qPCR to normalize the results. Normalized relative expression values were finally analysed according to the ΔΔCt method (Livak and Schmittgen 2001).

Almost all RNAi treated groups showed a significant reduction of the respective transcript compared to the untreated control (**Fig. S4**). However, RNAi targeting of *nex-1* resulted in a weak RNAi efficiency with a non-significant decrease of the targeted transcript. Furthermore, RT-qPCR could not reveal a downregulation of *nex-2 via* RNAi at all. It is possible that the RNAi did not work properly, or that an unsuitable location of the qPCR primer led to a false negative result as explained in Shepard *et al.* (2005).

References

Livak KJ, Schmittgen TD (2001) Analysis of relative gene expression data using real-time quantitative PCR and the 2(-Delta Delta C(T)) Method. Methods 25:402-408. doi:10.1006/meth.2001.1262

Ly K, Reid SJ, Snell RG (2015) Rapid RNA analysis of individual Caenorhabditis elegans. MethodsX 2:59-63. doi:10.1016/j.mex.2015.02.002

Shepard AR, Jacobson N, Clark AF (2005) Importance of quantitative PCR primer location for short interfering RNA efficacy determination. Anal Biochem 344:287-288. doi:10.1016/j.ab.2005.06.005

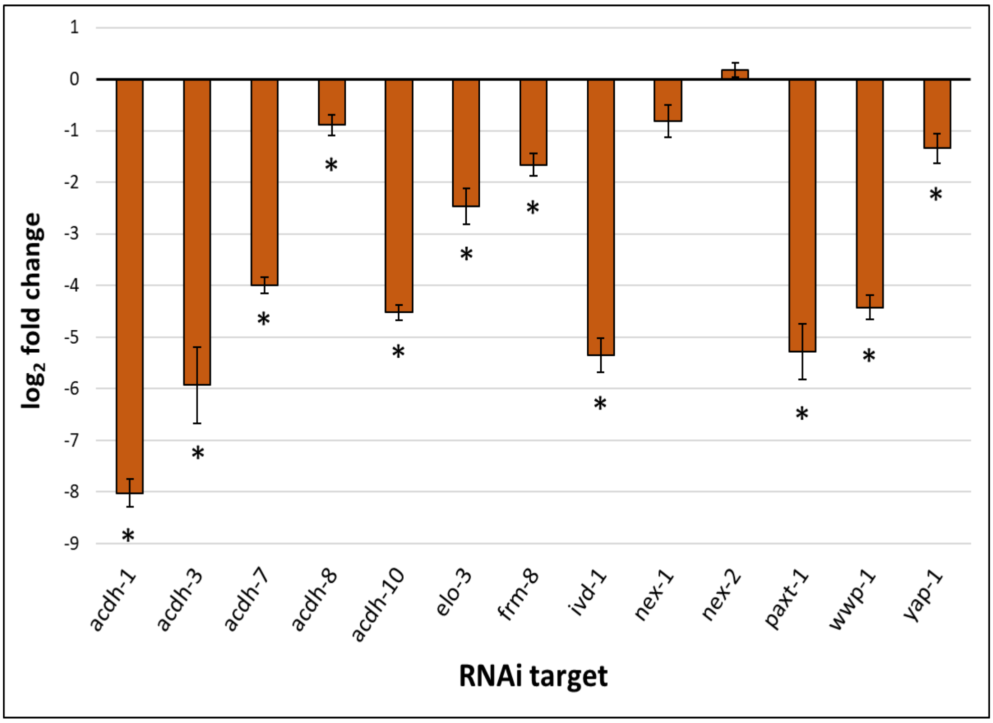

Fig S4: Verification of RNAi efficiency via RT-qPCR
Nematodes were grown until the 3rd day of adulthood and RNAi was triggered starting at the L4 stage. Normalization was done with the reference genes *act-1* and *cdc-42* and $\log_2$ fold changes are shown relative to the EV fed control. Primers were selected according to the RNAi target. Each bar represents three cDNA samples with four nematodes per sample. Each sample was measured in three technical replicates. Error bars show the standard error of the mean and differences compared to control were considered significant at $p < 0.05$ (*) determined in a student's t-test.

Table S1: Used primer pairs for RT-qPCR

Primer	5'→3' sequence		Annealing temperature
act-1	fw	TCCAAGAGAGGTATCCTTAC	56 °C
	rv	CGGTTAGCCTTTGGATTGAG	
cdc-42	fw	ATTACGCCGTCACAGTAATG	56 °C
	rv	ATCCCTGAGATCGACTTGAG	
acdh-1	fw	TGGGAGAGTATGGAAAAGGCTAC	56 °C
	rv	AAACAACCCTGAGCCAGTCC	
acdh-3	fw	CGTACCATCACCACTGCAAC	60 °C
	rv	TGCTCCGATTCCGATACGTC	
acdh-7	fw	AGAGTGGTGCAGAGGTTGAC	56 °C
	rv	AAACGGGTGGCGTGCTAATA	
acdh-8	fw	AGTTGTTGCTGTCAAAAGTGC	60 °C
	rv	TGAGTGTCGGACAGTTCAAA	
acdh-10	fw	GGGAGGACATGCTAAATGGTTC	56 °C
	rv	CGACATCGTTGGCGGATTTG	
elo-3	fw	AGAAGGAAAAGGAAGAGCCTGT	56 °C
	rv	GTTTTGCGTGGCCTCTGATG	
frm-8	fw	CCCTCCGTACTCGGCAATAC	54 °C
	rv	GGAGTTGAAATTAAGTTTTCTCCCT	
ivd-1	fw	TCGGATCATTCCAGCTCCTC	60 °C
	rv	TTTAAGACGAGCGGGCCTTC	
nex-1	fw	TCAGGCCAAGGATGATGCTG	54 °C
	rv	ACAATCCAGTCTGGTACATATTAGG	
nex-2	fw	AGTACCCTCCACAGCAAGGA	54 °C
	rv	GAGACGAGGAGACGCTTGAA	
paxt-1	fw	ATGATGACTATCCGAAAATCCAAC	60 °C
	rv	GTCCACCGGAACTCCATCAA	
wwp-1	fw	CGTCTACTCAGCCATTGCCA	60 °C
	rv	TCGGAGCCTCTGAAACCCT	
yap-1	fw	GCAGGACTCTCGGAAATACCA	54 °C
	rv	TGCACCATTGATCCCACTCC	
