# Supplementary figures and images for: Identification of healthspan-promoting genes in Caenorhabditis elegans based on a human GWAS study
Source: Biogerontology. 2022 Jun 24;23(4):431–52. doi: 10.1007/s10522-022-09969-8 (PMC9388463; doi:10.1007/s10522-022-09969-8)

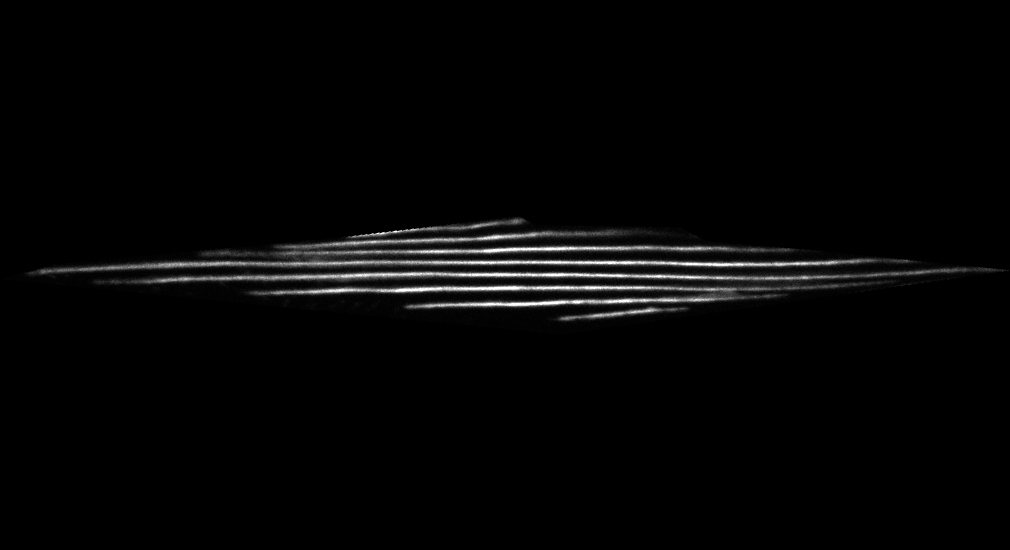

Supplement: Supplementary file 11 — Supplementary file11 (ZIP 217905 kb) [file 10522_2022_9969_MOESM11_ESM.zip › Images - supplementals/Repl 1/G02_w01_v03_Cropped.tif]

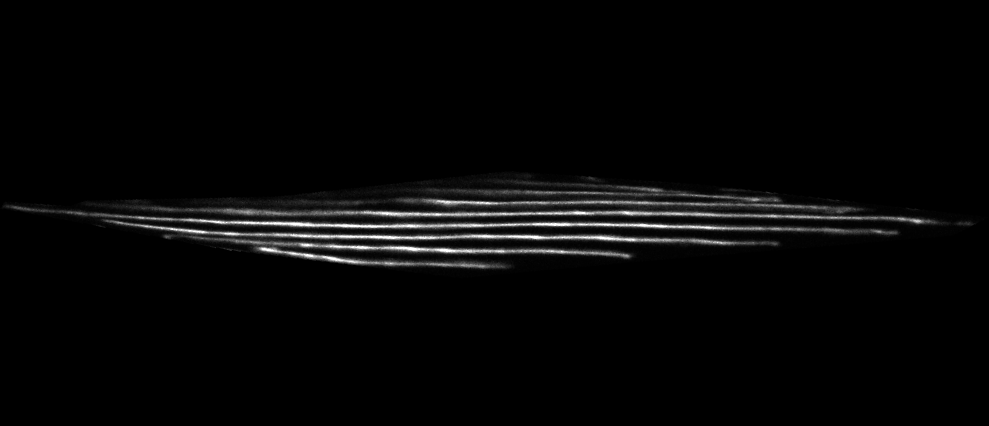

Supplement: Supplementary file 11 — Supplementary file11 (ZIP 217905 kb) [file 10522_2022_9969_MOESM11_ESM.zip › Images - supplementals/Repl 1/G02_w02_v01_Cropped.tif]

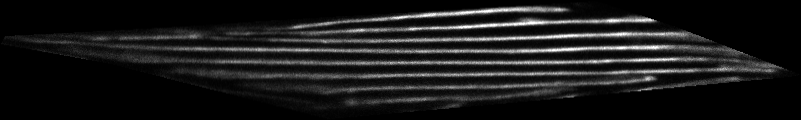

Supplement: Supplementary file 11 — Supplementary file11 (ZIP 217905 kb) [file 10522_2022_9969_MOESM11_ESM.zip › Images - supplementals/Repl 1/G02_w02_v03_Cropped.tif]

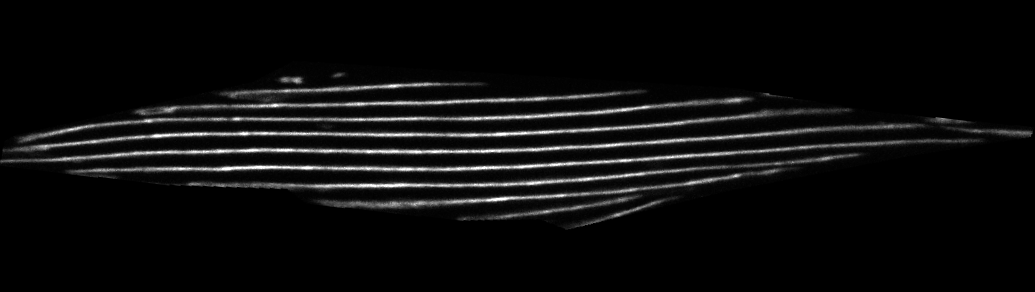

Supplement: Supplementary file 11 — Supplementary file11 (ZIP 217905 kb) [file 10522_2022_9969_MOESM11_ESM.zip › Images - supplementals/Repl 1/G02_w04_v02_Cropped.tif]

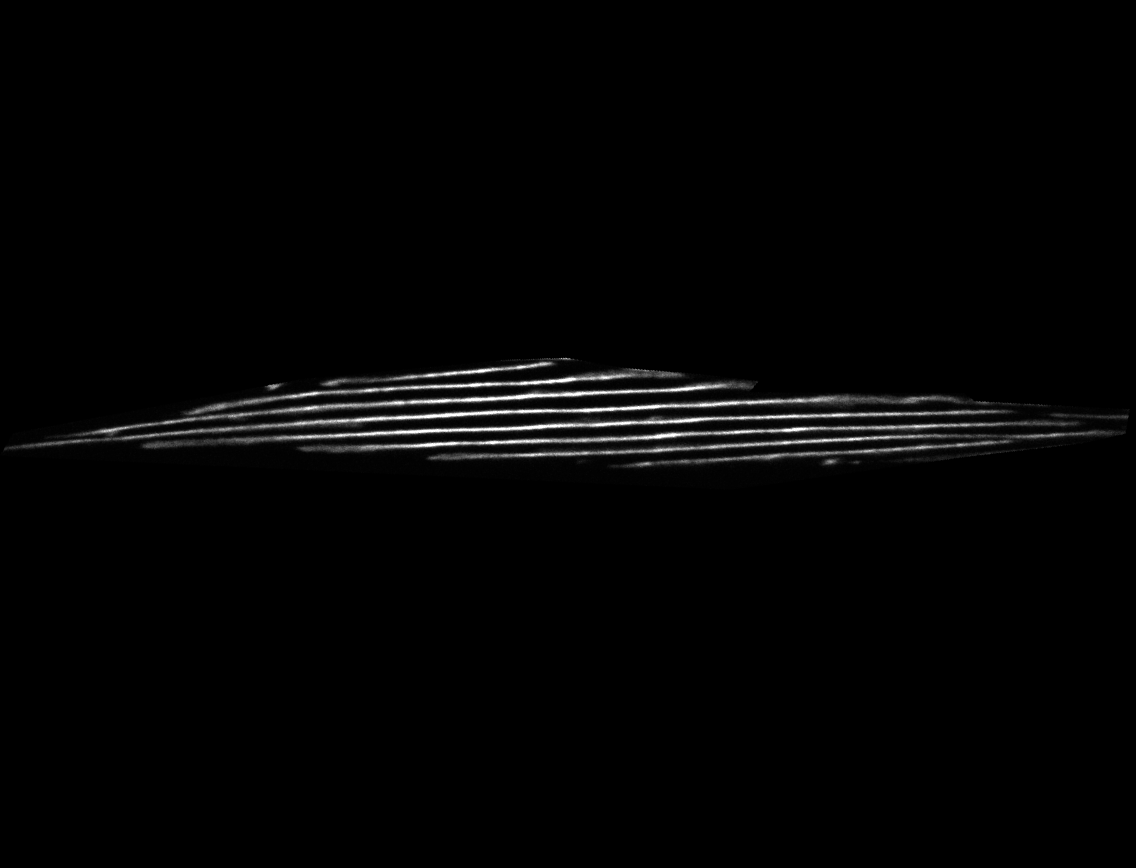

Supplement: Supplementary file 11 — Supplementary file11 (ZIP 217905 kb) [file 10522_2022_9969_MOESM11_ESM.zip › Images - supplementals/Repl 1/G02_w04_v03_Cropped.tif]

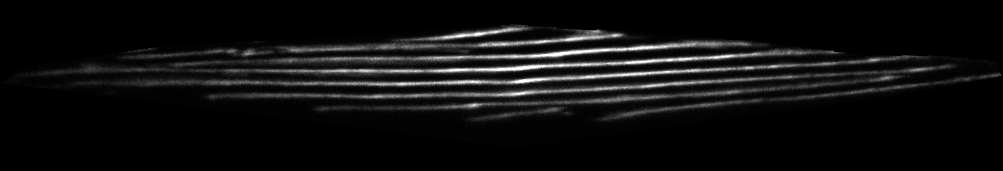

Supplement: Supplementary file 11 — Supplementary file11 (ZIP 217905 kb) [file 10522_2022_9969_MOESM11_ESM.zip › Images - supplementals/Repl 1/G02_w05_v02_Cropped.tif]

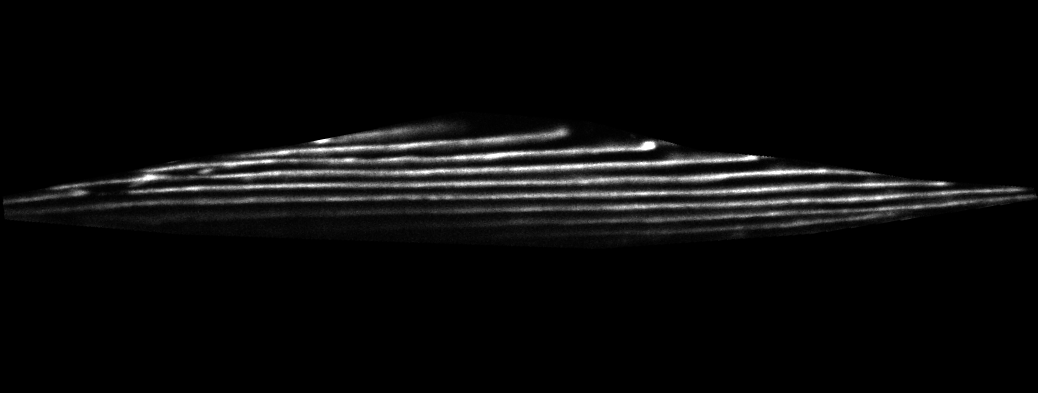

Supplement: Supplementary file 11 — Supplementary file11 (ZIP 217905 kb) [file 10522_2022_9969_MOESM11_ESM.zip › Images - supplementals/Repl 1/G02_w06_v01_Cropped.tif]

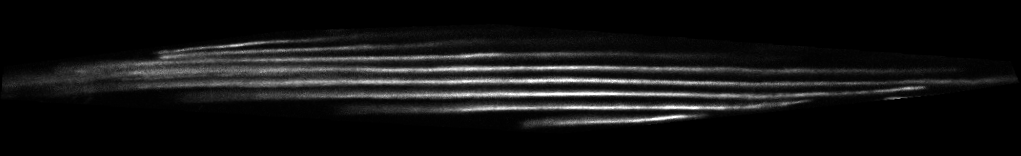

Supplement: Supplementary file 11 — Supplementary file11 (ZIP 217905 kb) [file 10522_2022_9969_MOESM11_ESM.zip › Images - supplementals/Repl 1/G02_w07_v01_Cropped.tif]

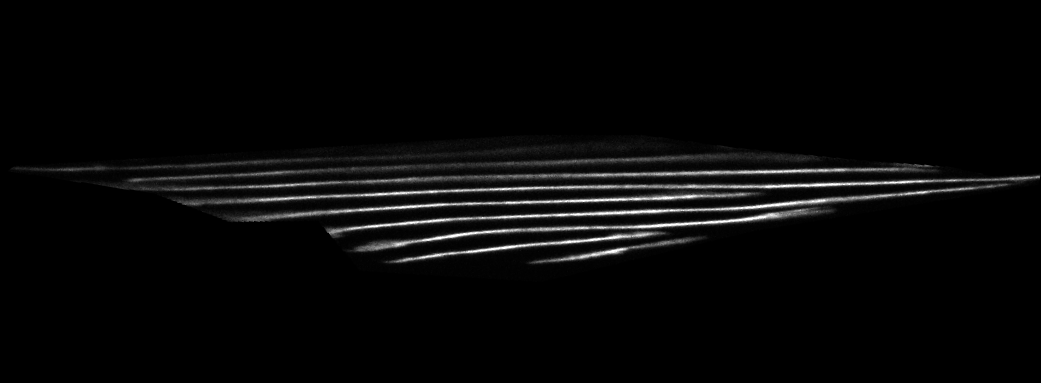

Supplement: Supplementary file 11 — Supplementary file11 (ZIP 217905 kb) [file 10522_2022_9969_MOESM11_ESM.zip › Images - supplementals/Repl 1/G03_w02_v01_Cropped.tif]

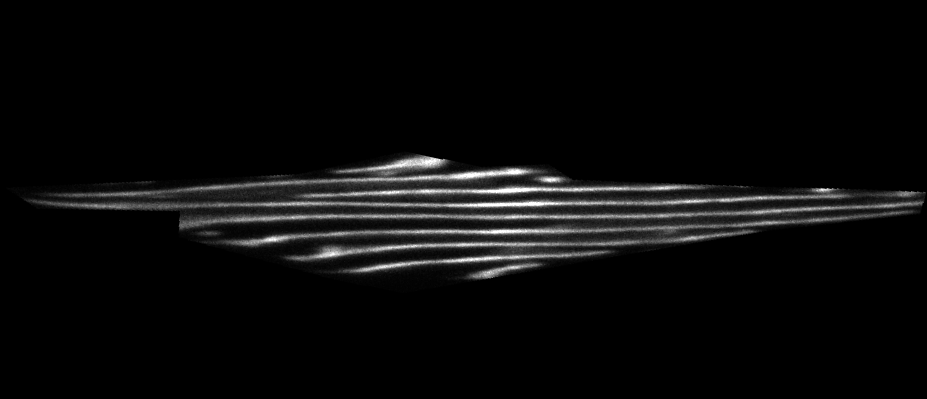

Supplement: Supplementary file 11 — Supplementary file11 (ZIP 217905 kb) [file 10522_2022_9969_MOESM11_ESM.zip › Images - supplementals/Repl 1/G03_w02_v02_Cropped.tif]

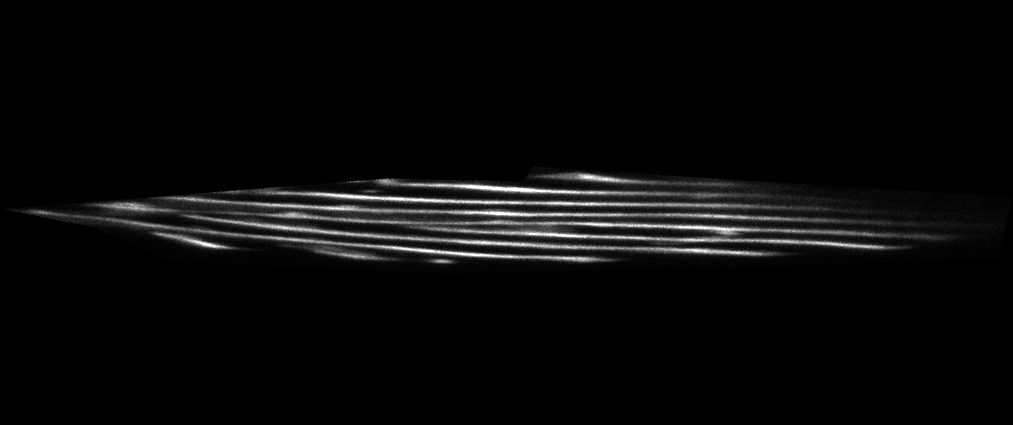

Supplement: Supplementary file 11 — Supplementary file11 (ZIP 217905 kb) [file 10522_2022_9969_MOESM11_ESM.zip › Images - supplementals/Repl 1/G03_w02_v03_Cropped.tif]

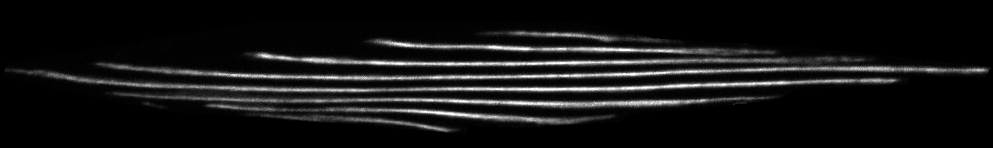

Supplement: Supplementary file 11 — Supplementary file11 (ZIP 217905 kb) [file 10522_2022_9969_MOESM11_ESM.zip › Images - supplementals/Repl 1/G03_w03_v01_Cropped.tif]

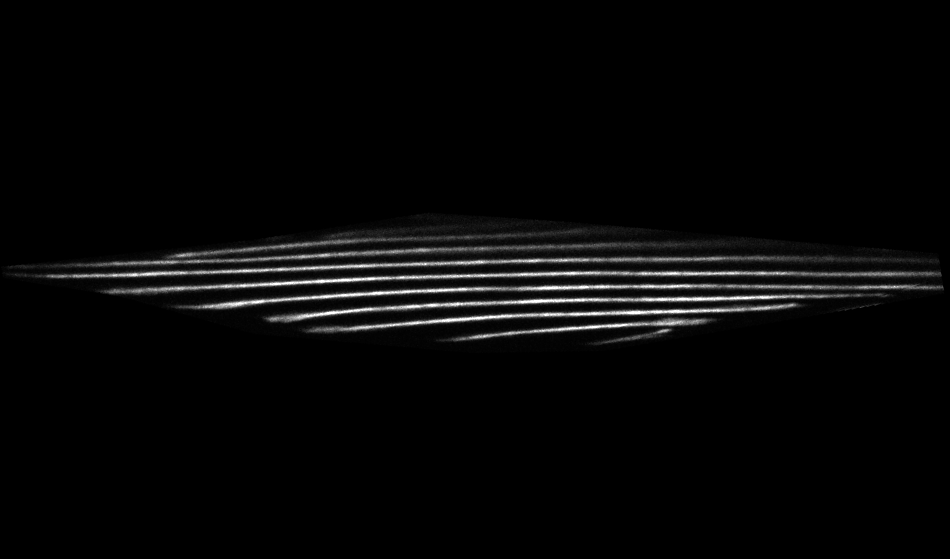

Supplement: Supplementary file 11 — Supplementary file11 (ZIP 217905 kb) [file 10522_2022_9969_MOESM11_ESM.zip › Images - supplementals/Repl 1/G03_w03_v02_Cropped.tif]

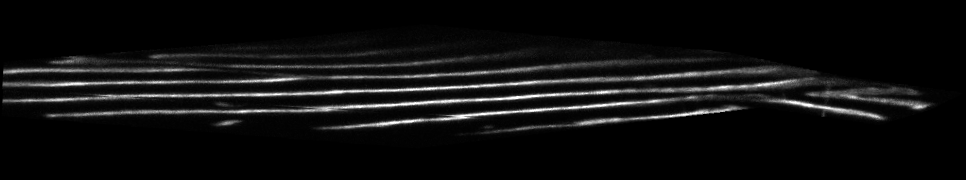

Supplement: Supplementary file 11 — Supplementary file11 (ZIP 217905 kb) [file 10522_2022_9969_MOESM11_ESM.zip › Images - supplementals/Repl 1/G03_w03_v03_Cropped.tif]

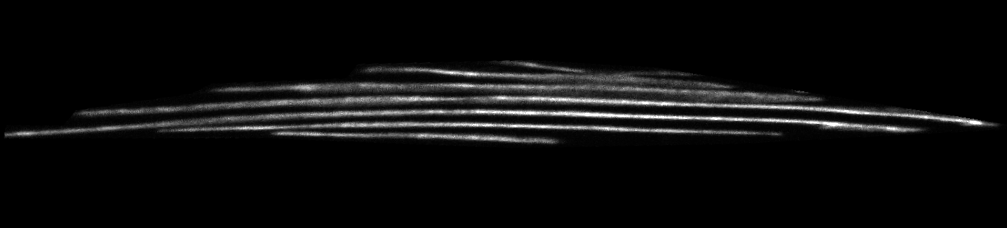

Supplement: Supplementary file 11 — Supplementary file11 (ZIP 217905 kb) [file 10522_2022_9969_MOESM11_ESM.zip › Images - supplementals/Repl 1/G03_w04_v01_Cropped.tif]

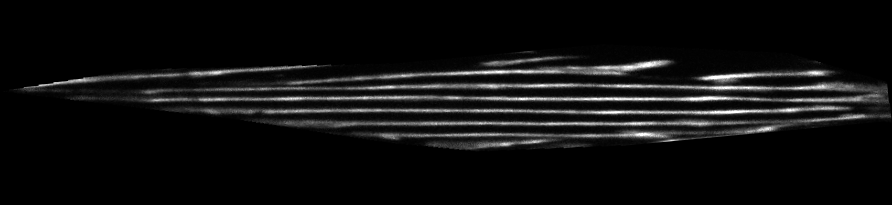

Supplement: Supplementary file 11 — Supplementary file11 (ZIP 217905 kb) [file 10522_2022_9969_MOESM11_ESM.zip › Images - supplementals/Repl 1/G03_w04_v03_Cropped.tif]

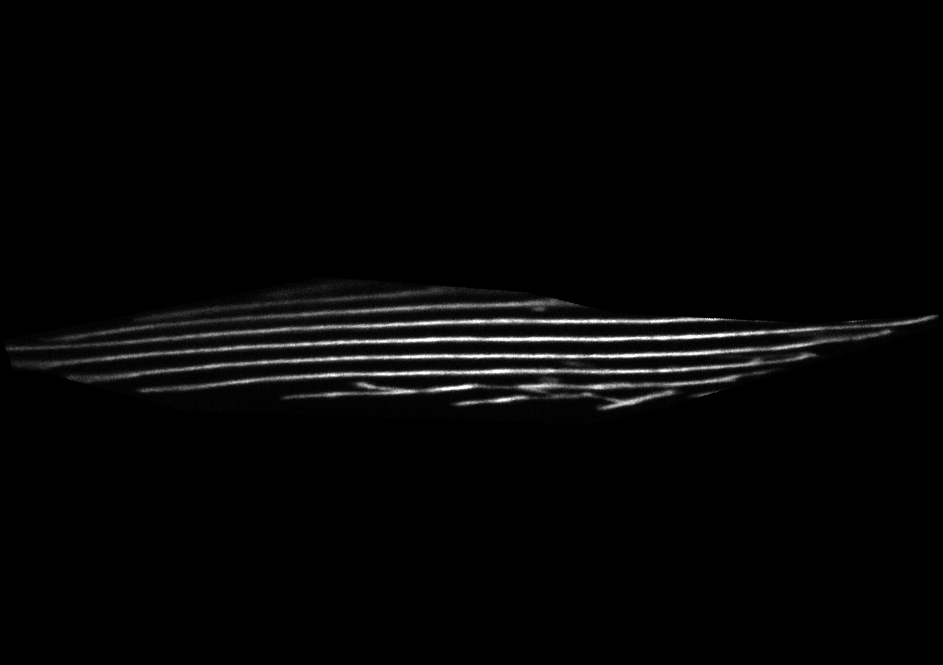

Supplement: Supplementary file 11 — Supplementary file11 (ZIP 217905 kb) [file 10522_2022_9969_MOESM11_ESM.zip › Images - supplementals/Repl 1/G03_w05_v01_Cropped.tif]

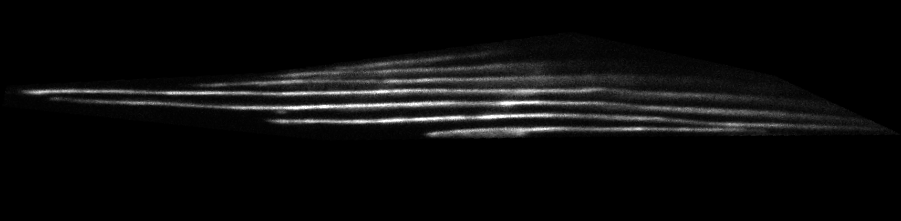

Supplement: Supplementary file 11 — Supplementary file11 (ZIP 217905 kb) [file 10522_2022_9969_MOESM11_ESM.zip › Images - supplementals/Repl 1/G03_w05_v03_Cropped.tif]

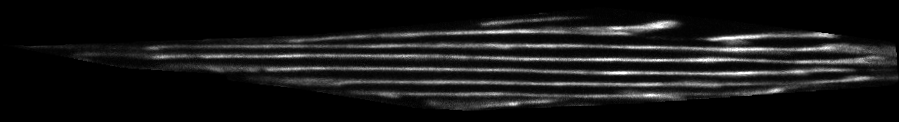

Supplement: Supplementary file 11 — Supplementary file11 (ZIP 217905 kb) [file 10522_2022_9969_MOESM11_ESM.zip › Images - supplementals/Repl 1/G03_w06_v04_Cropped.tif]

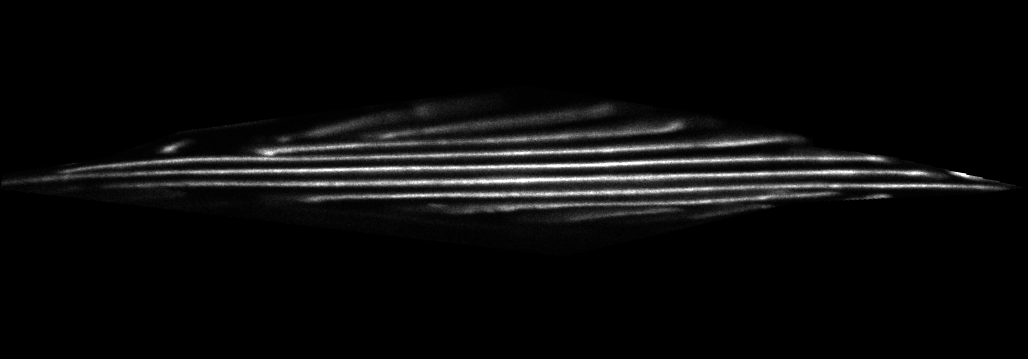

Supplement: Supplementary file 11 — Supplementary file11 (ZIP 217905 kb) [file 10522_2022_9969_MOESM11_ESM.zip › Images - supplementals/Repl 1/G03_w07_v01_Cropped.tif]

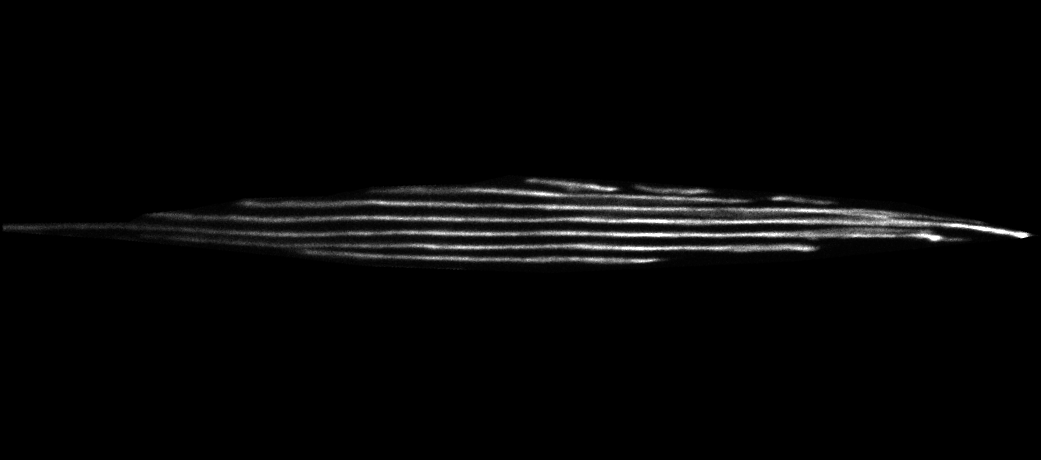

Supplement: Supplementary file 11 — Supplementary file11 (ZIP 217905 kb) [file 10522_2022_9969_MOESM11_ESM.zip › Images - supplementals/Repl 1/G03_w11_v01_Cropped.tif]

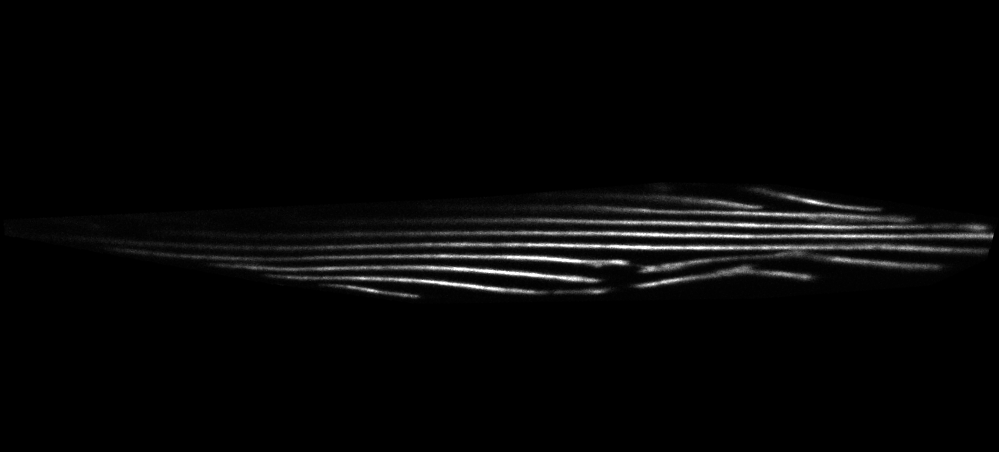

Supplement: Supplementary file 11 — Supplementary file11 (ZIP 217905 kb) [file 10522_2022_9969_MOESM11_ESM.zip › Images - supplementals/Repl 1/G07_w02_v02_Cropped.tif]

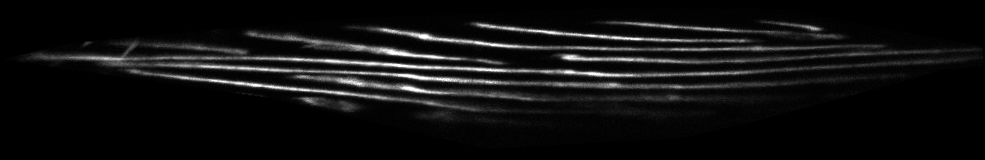

Supplement: Supplementary file 11 — Supplementary file11 (ZIP 217905 kb) [file 10522_2022_9969_MOESM11_ESM.zip › Images - supplementals/Repl 1/G07_w03_v01_Cropped.tif]

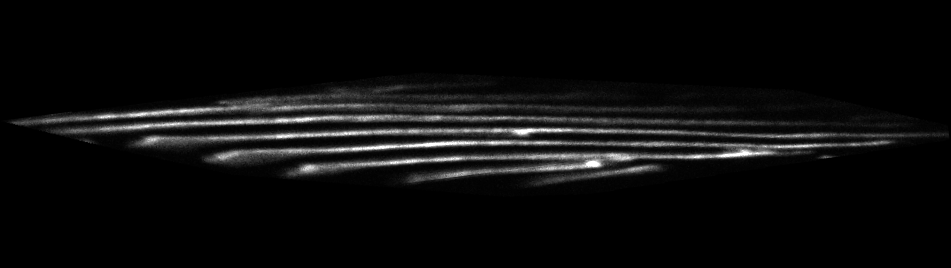

Supplement: Supplementary file 11 — Supplementary file11 (ZIP 217905 kb) [file 10522_2022_9969_MOESM11_ESM.zip › Images - supplementals/Repl 1/G07_w04_v01_Cropped.tif]

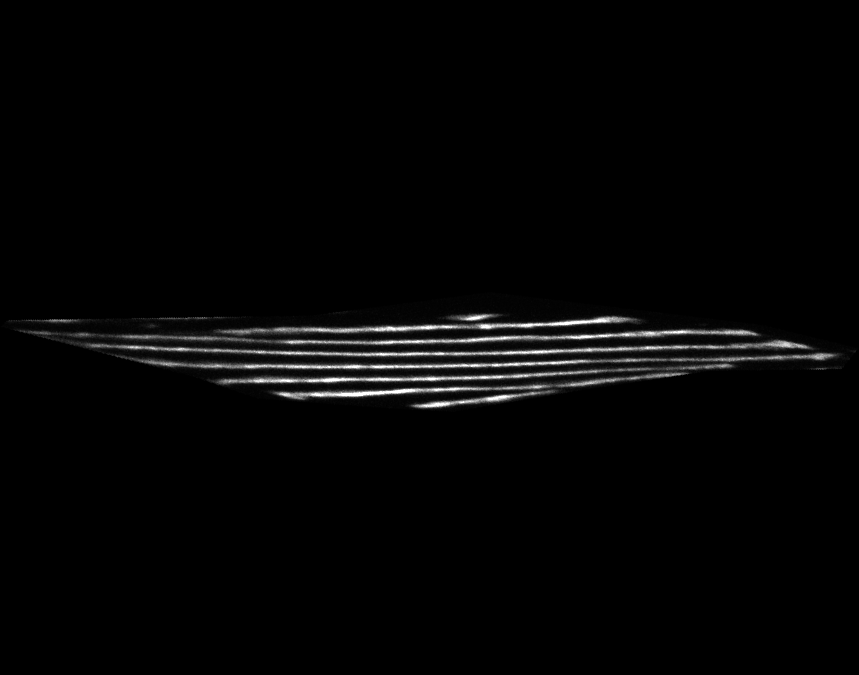

Supplement: Supplementary file 11 — Supplementary file11 (ZIP 217905 kb) [file 10522_2022_9969_MOESM11_ESM.zip › Images - supplementals/Repl 1/G07_w08_v03_Cropped.tif]

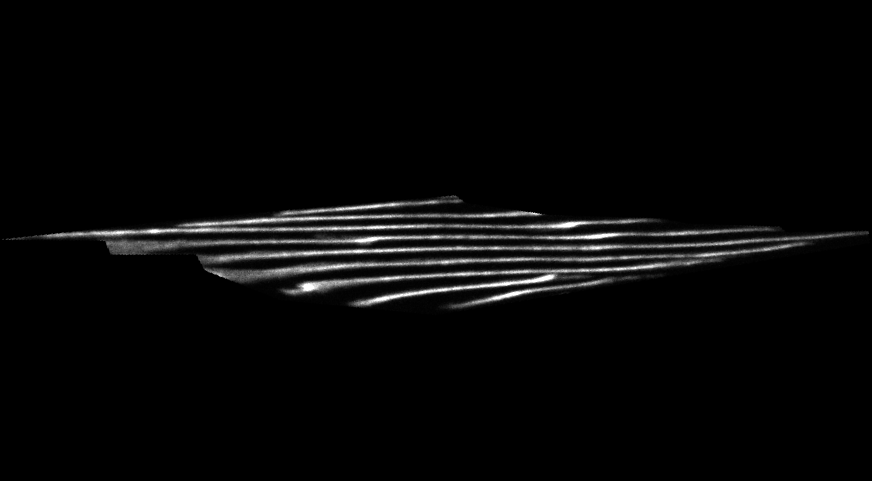

Supplement: Supplementary file 11 — Supplementary file11 (ZIP 217905 kb) [file 10522_2022_9969_MOESM11_ESM.zip › Images - supplementals/Repl 1/G07_w09_v02_Cropped.tif]

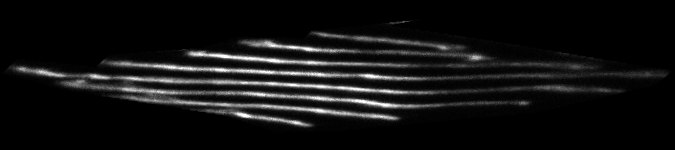

Supplement: Supplementary file 11 — Supplementary file11 (ZIP 217905 kb) [file 10522_2022_9969_MOESM11_ESM.zip › Images - supplementals/Repl 1/G08_w01_v01_02_Cropped.tif]

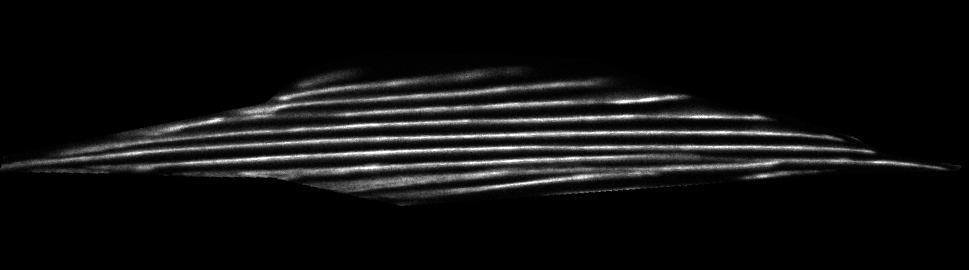

Supplement: Supplementary file 11 — Supplementary file11 (ZIP 217905 kb) [file 10522_2022_9969_MOESM11_ESM.zip › Images - supplementals/Repl 1/G08_w01_v03_Cropped.tif]

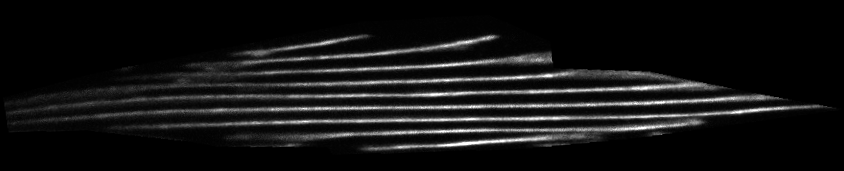

Supplement: Supplementary file 11 — Supplementary file11 (ZIP 217905 kb) [file 10522_2022_9969_MOESM11_ESM.zip › Images - supplementals/Repl 1/G08_w02_v01_Cropped.tif]

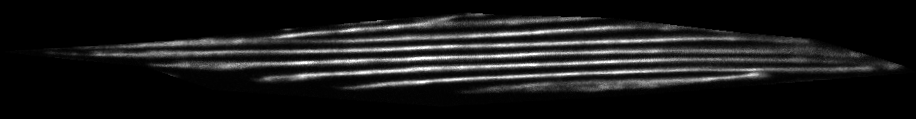

Supplement: Supplementary file 11 — Supplementary file11 (ZIP 217905 kb) [file 10522_2022_9969_MOESM11_ESM.zip › Images - supplementals/Repl 1/G08_w02_v02_Cropped.tif]

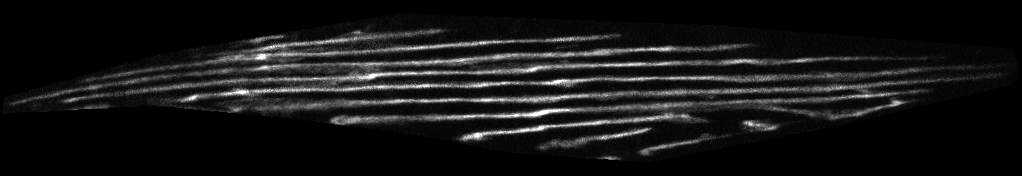

Supplement: Supplementary file 11 — Supplementary file11 (ZIP 217905 kb) [file 10522_2022_9969_MOESM11_ESM.zip › Images - supplementals/Repl 1/G08_w06_v02_Cropped.tif]

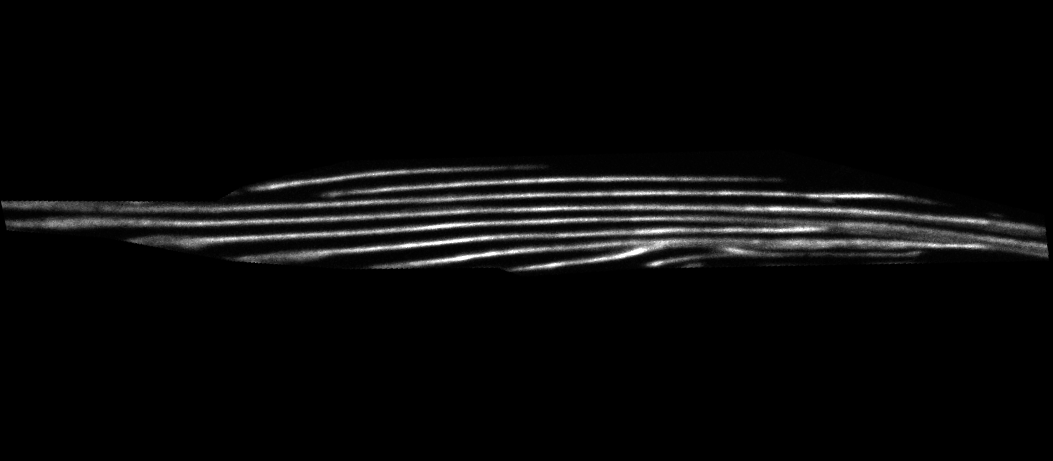

Supplement: Supplementary file 11 — Supplementary file11 (ZIP 217905 kb) [file 10522_2022_9969_MOESM11_ESM.zip › Images - supplementals/Repl 1/G09_w01_v03_Cropped.tif]

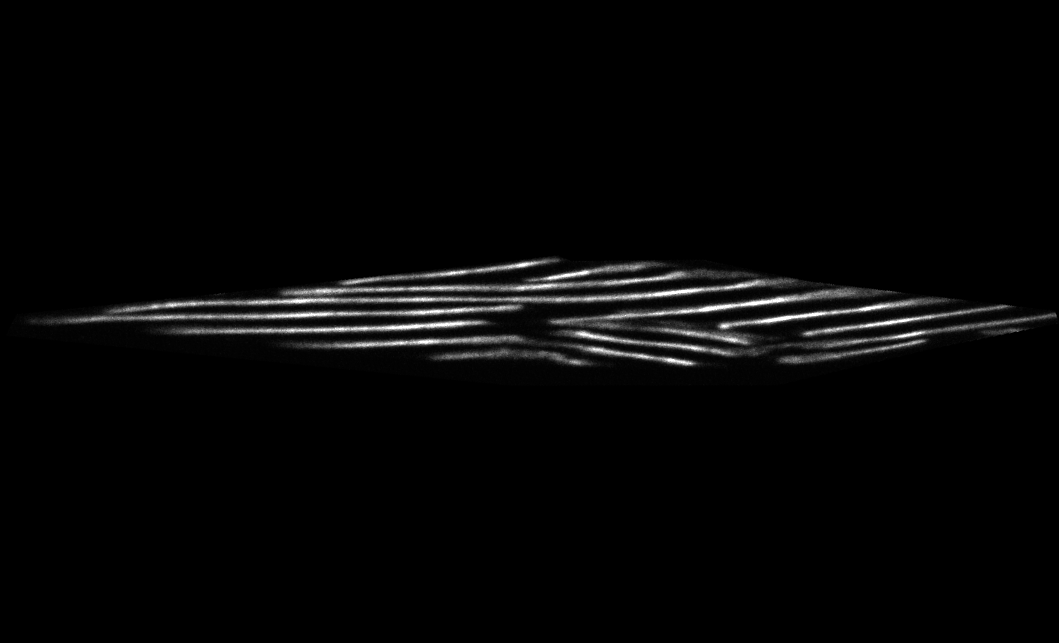

Supplement: Supplementary file 11 — Supplementary file11 (ZIP 217905 kb) [file 10522_2022_9969_MOESM11_ESM.zip › Images - supplementals/Repl 1/G09_w02_v03_Cropped.tif]

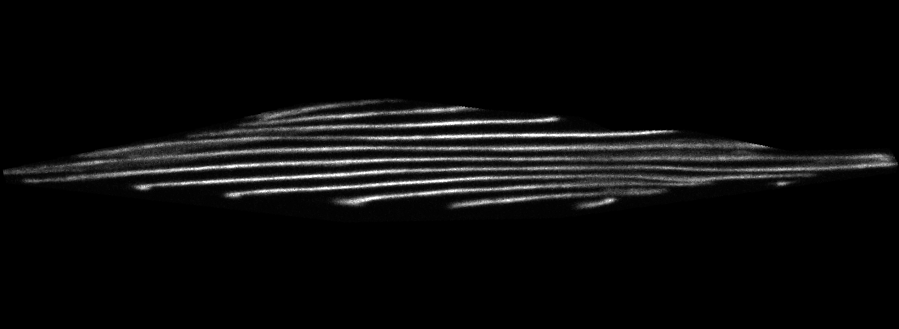

Supplement: Supplementary file 11 — Supplementary file11 (ZIP 217905 kb) [file 10522_2022_9969_MOESM11_ESM.zip › Images - supplementals/Repl 1/G09_w05_v02_Cropped.tif]

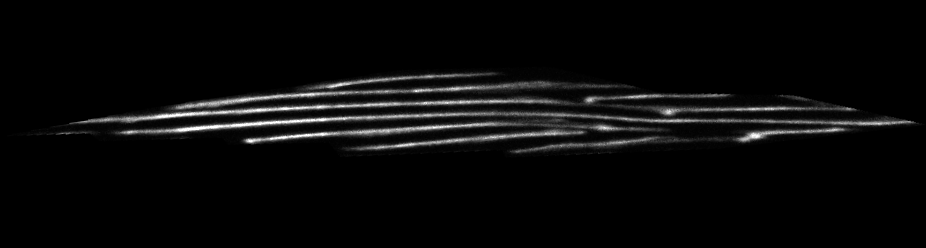

Supplement: Supplementary file 11 — Supplementary file11 (ZIP 217905 kb) [file 10522_2022_9969_MOESM11_ESM.zip › Images - supplementals/Repl 1/G09_w05_v03_Cropped.tif]

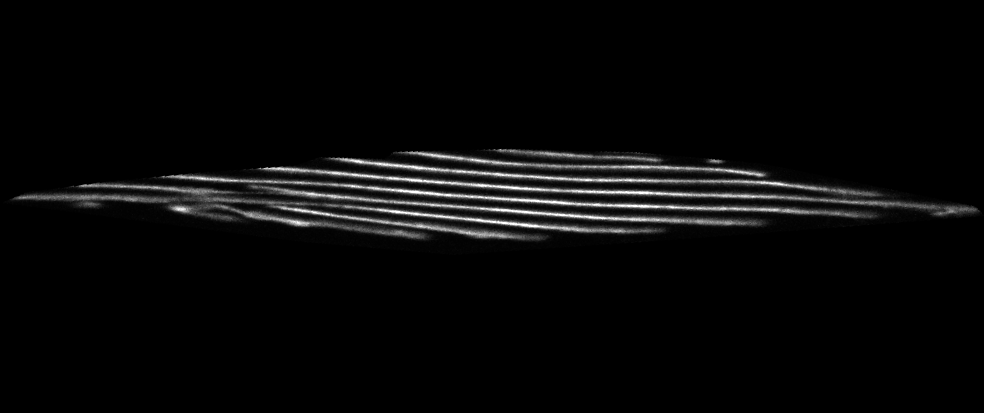

Supplement: Supplementary file 11 — Supplementary file11 (ZIP 217905 kb) [file 10522_2022_9969_MOESM11_ESM.zip › Images - supplementals/Repl 1/G09_w06_v01_Cropped.tif]

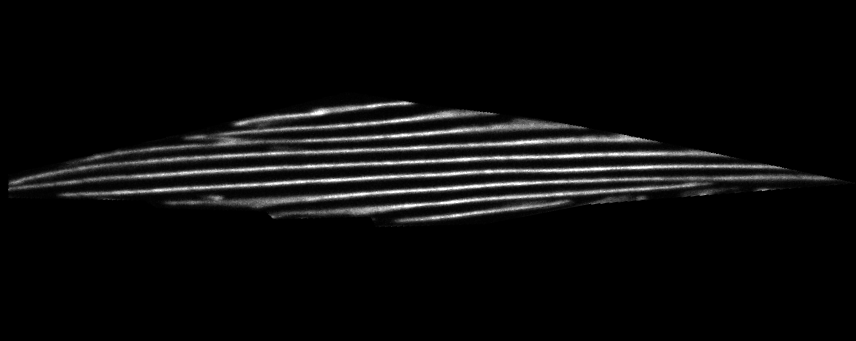

Supplement: Supplementary file 11 — Supplementary file11 (ZIP 217905 kb) [file 10522_2022_9969_MOESM11_ESM.zip › Images - supplementals/Repl 1/G09_w07_v03_Cropped.tif]

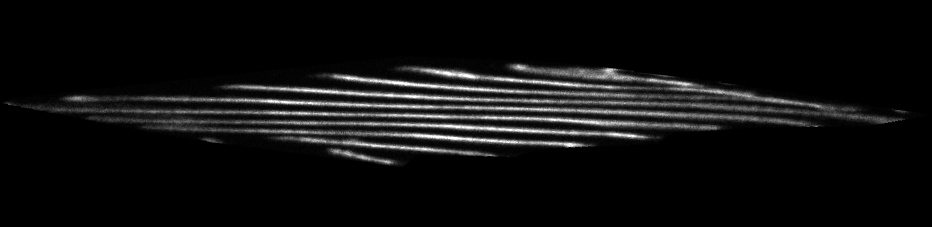

Supplement: Supplementary file 11 — Supplementary file11 (ZIP 217905 kb) [file 10522_2022_9969_MOESM11_ESM.zip › Images - supplementals/Repl 1/G09_w08_v02_Cropped.tif]

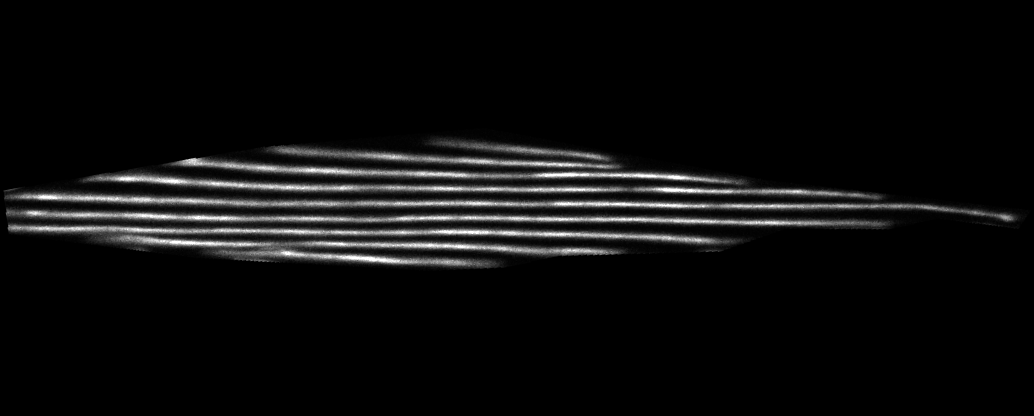

Supplement: Supplementary file 11 — Supplementary file11 (ZIP 217905 kb) [file 10522_2022_9969_MOESM11_ESM.zip › Images - supplementals/Repl 1/G10_w01_v01_Cropped.tif]

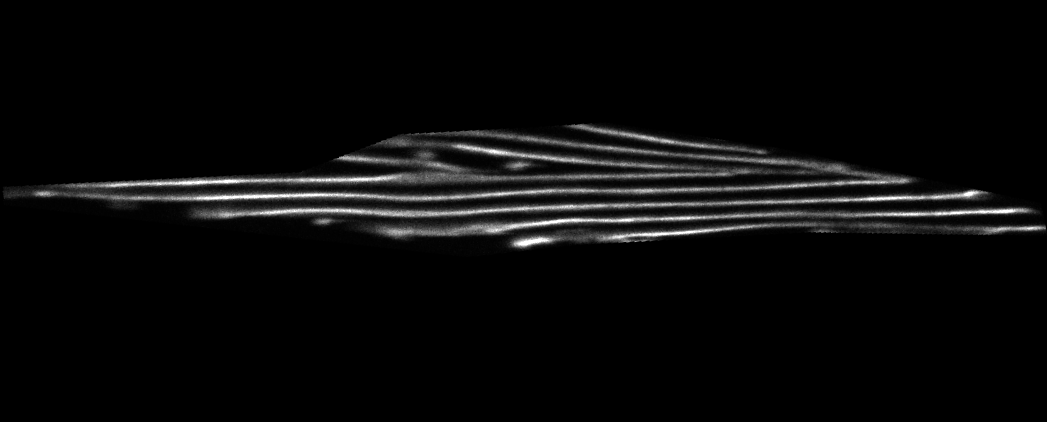

Supplement: Supplementary file 11 — Supplementary file11 (ZIP 217905 kb) [file 10522_2022_9969_MOESM11_ESM.zip › Images - supplementals/Repl 1/G10_w01_v02_Cropped.tif]

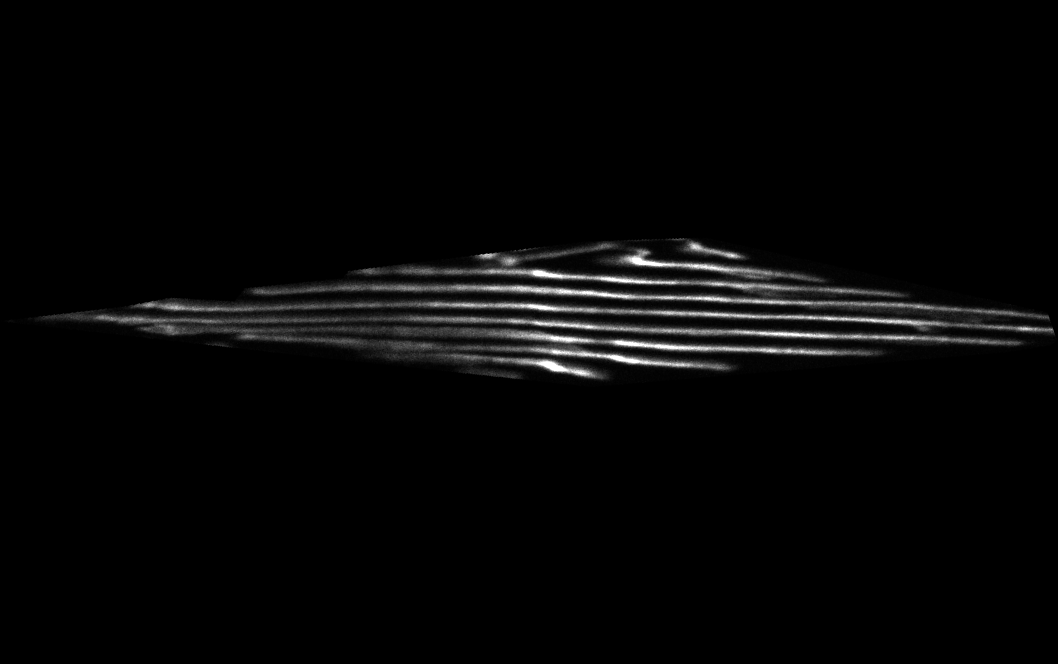

Supplement: Supplementary file 11 — Supplementary file11 (ZIP 217905 kb) [file 10522_2022_9969_MOESM11_ESM.zip › Images - supplementals/Repl 1/G10_w01_v03_Cropped.tif]

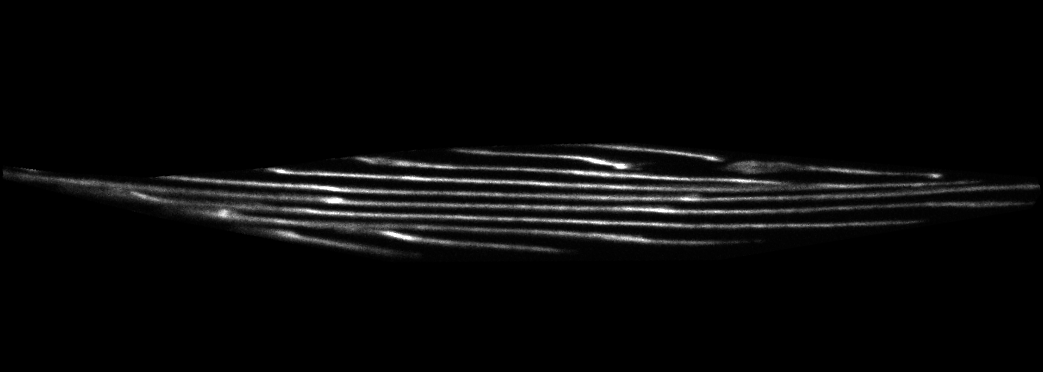

Supplement: Supplementary file 11 — Supplementary file11 (ZIP 217905 kb) [file 10522_2022_9969_MOESM11_ESM.zip › Images - supplementals/Repl 1/G10_w03_v02_Cropped.tif]

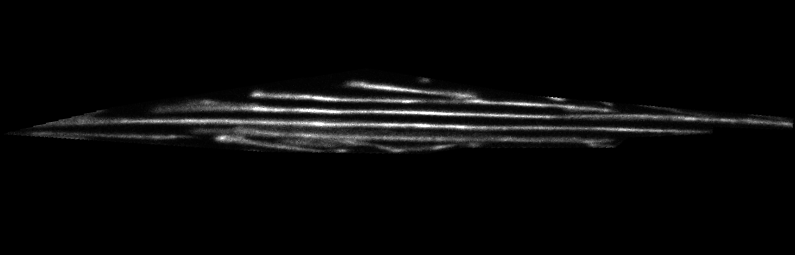

Supplement: Supplementary file 11 — Supplementary file11 (ZIP 217905 kb) [file 10522_2022_9969_MOESM11_ESM.zip › Images - supplementals/Repl 1/G10_w04_v01_Cropped.tif]

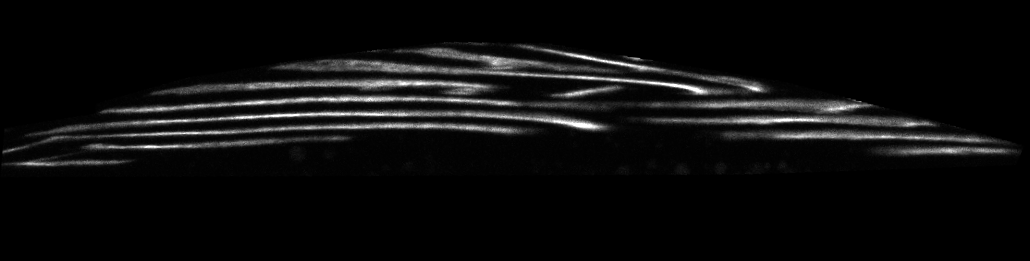

Supplement: Supplementary file 11 — Supplementary file11 (ZIP 217905 kb) [file 10522_2022_9969_MOESM11_ESM.zip › Images - supplementals/Repl 1/G10_w05_v02_Cropped.tif]

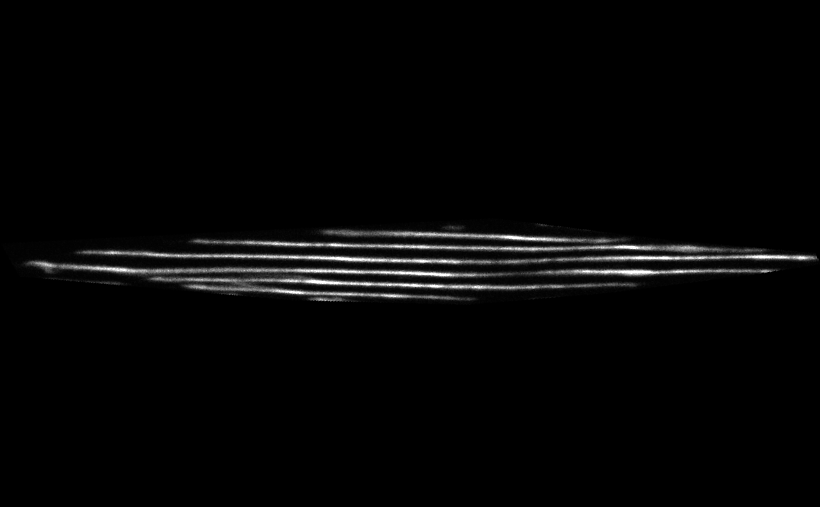

Supplement: Supplementary file 11 — Supplementary file11 (ZIP 217905 kb) [file 10522_2022_9969_MOESM11_ESM.zip › Images - supplementals/Repl 1/G10_w06_v03_Cropped.tif]

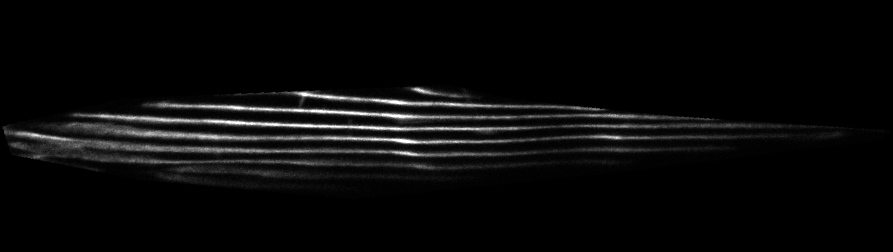

Supplement: Supplementary file 11 — Supplementary file11 (ZIP 217905 kb) [file 10522_2022_9969_MOESM11_ESM.zip › Images - supplementals/Repl 1/G10_w07_v01_Cropped.tif]

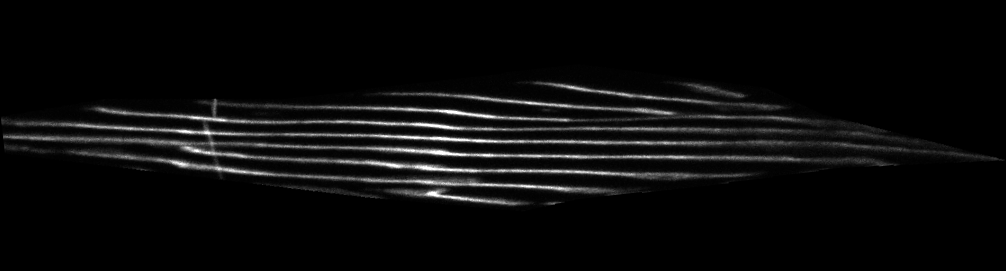

Supplement: Supplementary file 11 — Supplementary file11 (ZIP 217905 kb) [file 10522_2022_9969_MOESM11_ESM.zip › Images - supplementals/Repl 1/G10_w07_v02_Cropped.tif]

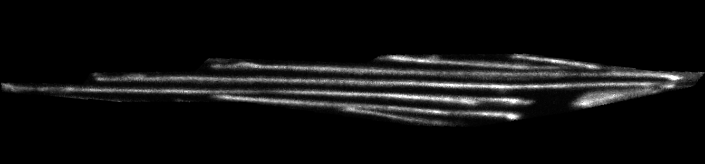

Supplement: Supplementary file 11 — Supplementary file11 (ZIP 217905 kb) [file 10522_2022_9969_MOESM11_ESM.zip › Images - supplementals/Repl 1/G10_w08_v03_Cropped.tif]

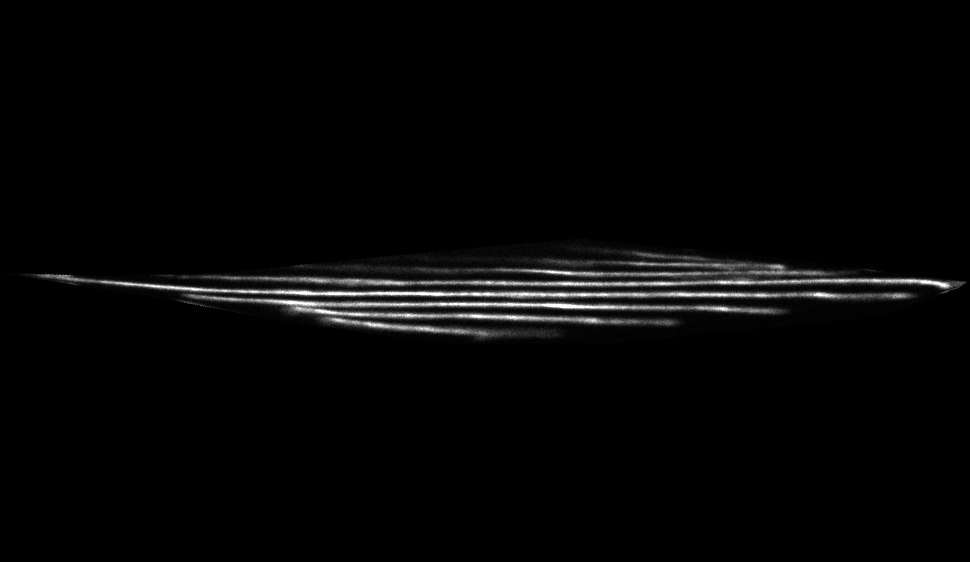

Supplement: Supplementary file 11 — Supplementary file11 (ZIP 217905 kb) [file 10522_2022_9969_MOESM11_ESM.zip › Images - supplementals/Repl 1/G11_w01_v01_Cropped.tif]

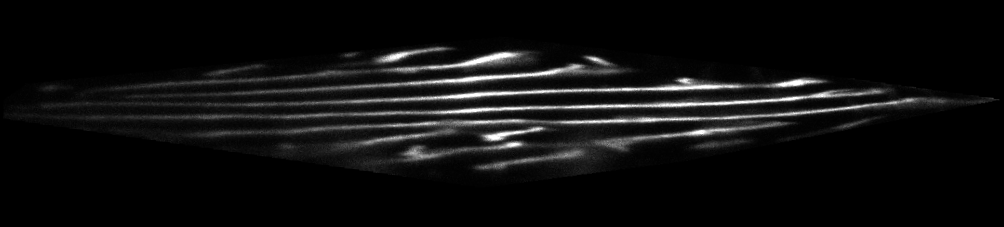

Supplement: Supplementary file 11 — Supplementary file11 (ZIP 217905 kb) [file 10522_2022_9969_MOESM11_ESM.zip › Images - supplementals/Repl 1/G11_w09_v01_Cropped.tif]

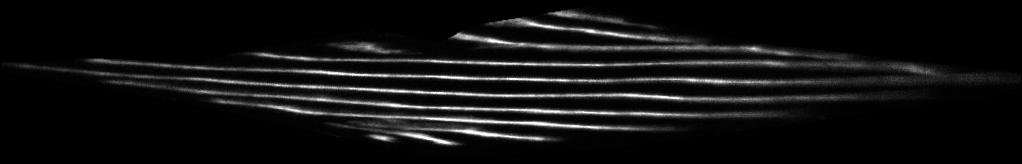

Supplement: Supplementary file 11 — Supplementary file11 (ZIP 217905 kb) [file 10522_2022_9969_MOESM11_ESM.zip › Images - supplementals/Repl 1/G12_w01_v03_Cropped.tif]

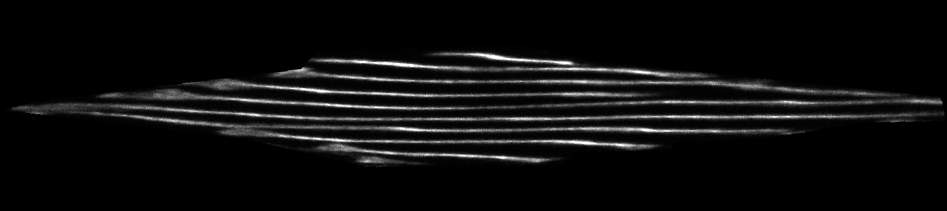

Supplement: Supplementary file 11 — Supplementary file11 (ZIP 217905 kb) [file 10522_2022_9969_MOESM11_ESM.zip › Images - supplementals/Repl 1/G12_w02_v01_Cropped.tif]

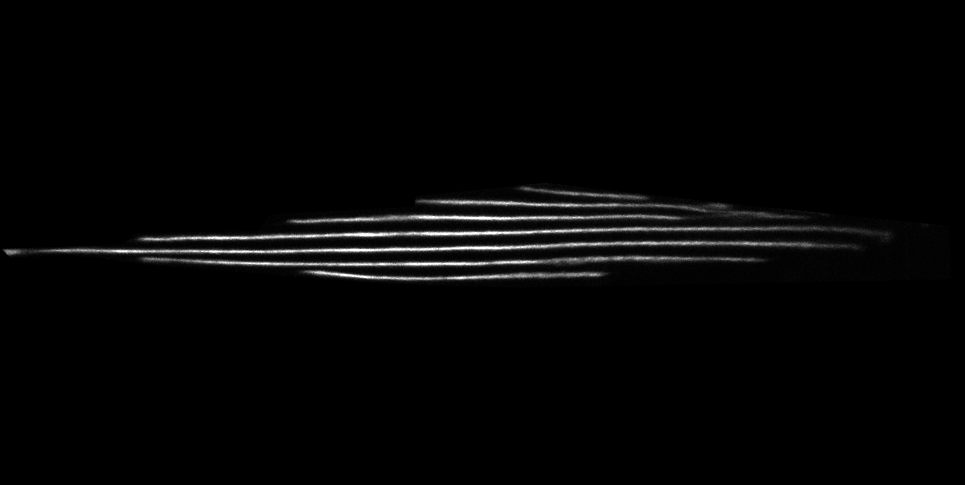

Supplement: Supplementary file 11 — Supplementary file11 (ZIP 217905 kb) [file 10522_2022_9969_MOESM11_ESM.zip › Images - supplementals/Repl 1/G12_w03_v01_Cropped.tif]

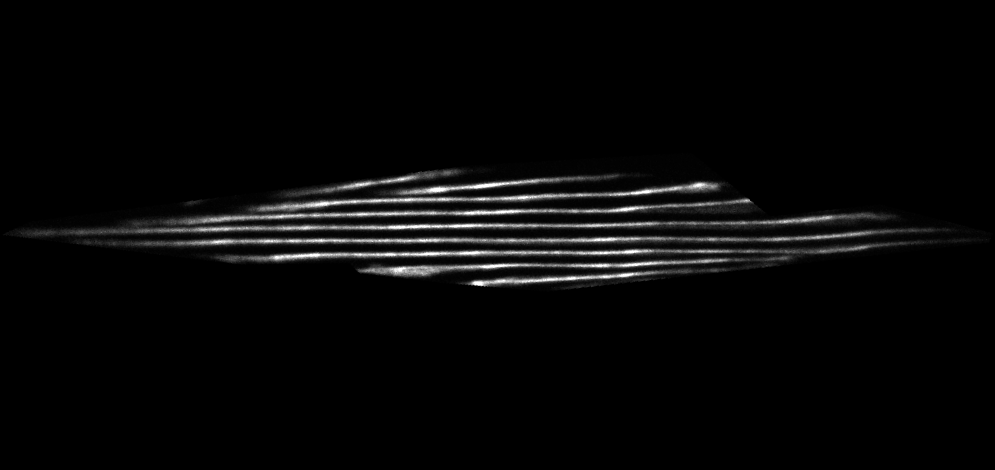

Supplement: Supplementary file 11 — Supplementary file11 (ZIP 217905 kb) [file 10522_2022_9969_MOESM11_ESM.zip › Images - supplementals/Repl 1/G12_w03_v03_Cropped.tif]

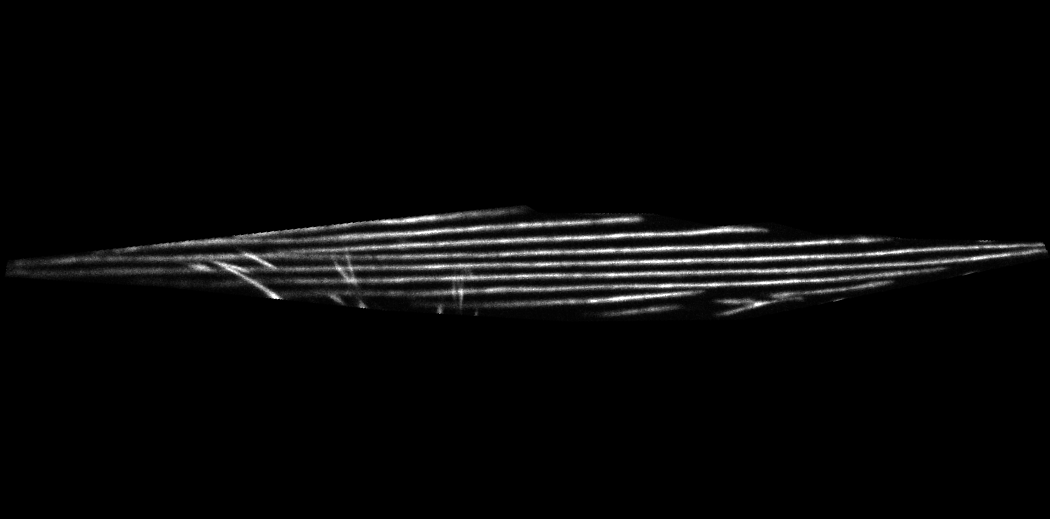

Supplement: Supplementary file 11 — Supplementary file11 (ZIP 217905 kb) [file 10522_2022_9969_MOESM11_ESM.zip › Images - supplementals/Repl 1/G12_w04_v03_Cropped.tif]

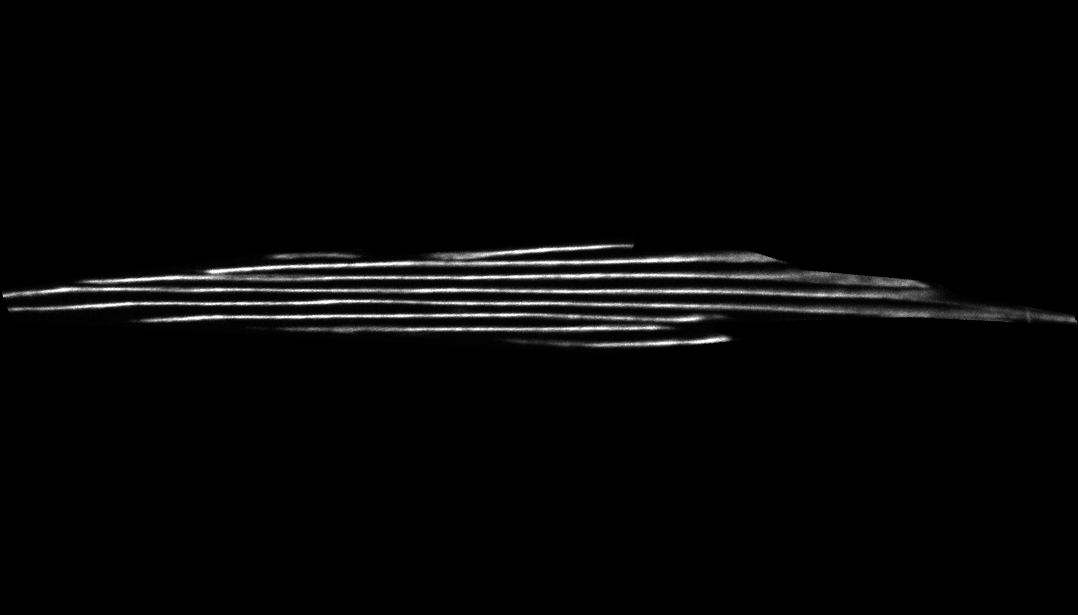

Supplement: Supplementary file 11 — Supplementary file11 (ZIP 217905 kb) [file 10522_2022_9969_MOESM11_ESM.zip › Images - supplementals/Repl 1/G12_w06_v01_Cropped.tif]

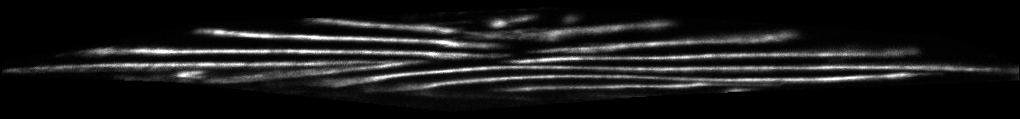

Supplement: Supplementary file 11 — Supplementary file11 (ZIP 217905 kb) [file 10522_2022_9969_MOESM11_ESM.zip › Images - supplementals/Repl 1/G12_w08_v02_Cropped.tif]

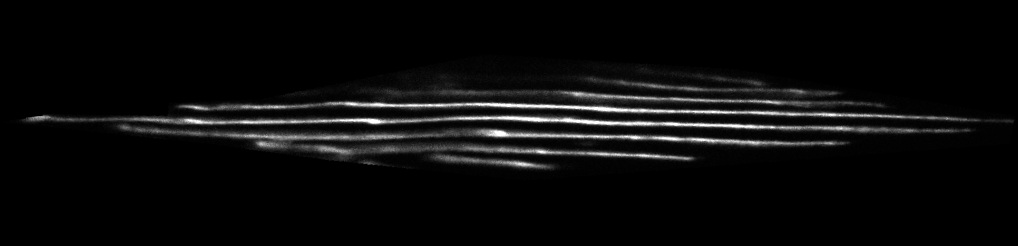

Supplement: Supplementary file 11 — Supplementary file11 (ZIP 217905 kb) [file 10522_2022_9969_MOESM11_ESM.zip › Images - supplementals/Repl 1/G12_w09_v02_Cropped.tif]

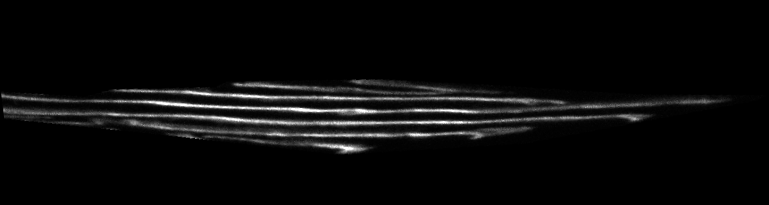

Supplement: Supplementary file 11 — Supplementary file11 (ZIP 217905 kb) [file 10522_2022_9969_MOESM11_ESM.zip › Images - supplementals/Repl 1/G12_w10_v02_Cropped.tif]
